# Neighbourhood Sum Degree-Based Indices and Entropy Measures for Certain Family of Graphene Molecules

**DOI:** 10.3390/molecules28010168

**Published:** 2022-12-25

**Authors:** Jun Yang, Julietraja Konsalraj, Arul Amirtha Raja S.

**Affiliations:** 1 School of Economics and Law, Chaohu University, Chaohu 238000, China; 2 Department of Mathematics, St. Joseph’s College of Engineering, OMR, Chennai 600119, India

**Keywords:** degree-based TIs, M-Polynomial, neighbourhood sum degree-based indices, entropy measures

## Abstract

A topological index (TI) is a real number that defines the relationship between a chemical structure and its properties and remains invariant under graph isomorphism. TIs defined for chemical structures are capable of predicting physical properties, chemical reactivity and biological activity. Several kinds of TIs have been defined and studied for different molecular structures. Graphene is the thinnest material known to man and is also extremely strong while being a good conductor of heat and electricity. With such unique features, graphene and its derivatives have found commercial uses and have also fascinated theoretical chemists. In this article, the neighbourhood sum degree-based M-polynomial and entropy measures have been computed for graphene, graphyne and graphdiyne structures. The proper analytical expressions for these indices are derived. The obtained results will enable theoretical chemists to study these exciting structures further from a structural perspective.

## 1. Introduction

The study of the association between the structural formula of chemical compounds and their corresponding topological graphs was a ground-breaking advancement in investigating chemical structures. Since then, the basic concepts of chemical graph theory have been developed as a combination of principles from both mathematics and chemistry. Chemical graph theory enables mathematicians and chemists to theoretically analyse molecular compounds by depicting their structures as graphs. The vertices represent the atoms of molecules or molecule collections in a chemical graph. The edges of a chemical graph indicate the relationships between the chemical objects and generally represent reactions, reaction mechanisms, chemical bonds, or other transformations in chemical entities.

Graph theoretical tools have been gaining popularity as the primary techniques for the theoretical study of chemical compounds. QSAR/QSPR techniques, in particular, are regarded as useful computational and quasi-strategies for trying to predict the characteristics of chemical substances. These techniques are crucial in the development of new and more effective herbicides because their properties can be estimated prior to synthesising and thus influence the design. Furthermore, experimental measurements can be replaced by QSPR/QSAR models, which are less expensive and time-consuming [1]. In this context, Topological indices provide a quantitative characterisation of molecular topology.

A topological index (TI) is a real number that defines the relationship between a chemical structure and its properties and remains invariant under graph isomorphism. TIs defined for chemical structures are capable of predicting physical properties, chemical reactivity, and biological activity. Several kinds of TIs have been defined and studied for different molecular structures. Graphene is the thinnest material known to man and is also extremely strong while being a good conductor of heat and electricity. With such unique features, graphene and its derivatives have found commercial uses and have also fascinated theoretical chemists.

The first occurrence of a topological index in the literature is in the pioneering work of the eminent chemist Wiener. He described the boiling point of alkanes in terms of a path number W, which is the sum of the distances between any two atoms within the molecule. This path number later became known as the Wiener index and has been studied extensively [2]. This ground-breaking work kickstarted the research on topological indices, and its mathematical investigation began in the 1970s. Since the behaviour and properties of molecules rely heavily on the corresponding molecular structures, TIs have now been established as major molecular indices in theoretical chemistry.

A considerable number of topological indices have been proven to display a strong correlation with several properties of chemical compounds. Due to their ability to characterise a large variety of physical and chemical properties, the study of topological indices has wide-ranging applications in various fields, including computer-assisted drug discovery, deriving multi-linear regression models [3], aromatic sextet theory [4], and thermochemistry [5]. The comparative ease of using molecular TIs to determine the physicochemical properties as opposed to the complex quantum chemical calculations has also found several applications for these TIs [6]. Thus, it becomes vital to determine the various molecular indices of molecules so that suitable indices are applied to attain the desired correlations between their properties and structures. There are several kinds of TIs that are currently defined and studied in the literature. The most commonly used TIs among them are distance and degree-based indices. A topological index is said to be distance-based if its computation involves distance between vertices. A degree-based topological index is a sub-class of TIs where the index is computed based on the degrees of the end vertices of the molecular graph.

One of the most efficient and convenient ways to compute TIs is by using algebraic polynomials, where the estimations of multiple TIs are reduced to the computation of a single polynomial. For example, the Hosoya polynomial [7] is widely applied for calculating distance-based TIs. Deutsch and Klavžar [8] created a similar polynomial for computing degree-based indices. In this technique, a single polynomial, referred to as the M-polynomial, is used to compute several prominent degree-based indices. A significant number of articles in the literature deal with M-polynomial and the computation of degree-based indices using it. The degree-based entropy measures are computed for these structures [9]. 

The neighbourhood sum degree-based TIs are one of the recent developments in analysing chemical compounds using graph theory. They have been used for QSPR and statistics regression analysis for predicting specific chemical properties of molecular graphs [10,11]. The degree-based entropy measures studied and widely applied in graph theory are considered information functionals in the study of networks. The applications of entropy network measures range from the description of the structure of a molecule quantitatively to the exploration of biological and chemical features of molecular graphs. Presently, very few articles in the literature demonstrate the computation of neighbourhood sum degree-based M-Polynomial and neighbourhood sum degree-based entropy measures. Hence, this article is primarily concerned with computing the neighbourhood sum degree-based M-Polynomial and neighbourhood sum degree-based entropy measures for three prominent structures: graphene, graphyne and graphdiyne. This computation has not been conducted for these structures yet. Therefore, the computed results will provide a unique viewpoint for researchers to study these structures. The findings can also be extended to other large structures with a similar structural base.

## 2. Results

### 2.1. Mathematical Concepts

Throughout this research, we consider only a simple and connected graph without multiple edges and self-loops. The graph Γ is said to be a connected graph with vertex set V(Γ) and edge set E(Γ). The degree of the vertex is represented as $d_{v}$.

### 2.2. Neighbourhood Degree Sum-Based Indices

The neighbourhood sum degree-based TIs are denoted by $N_{\Gamma }\left(v\right)$. The neighbourhood sum degree of the molecular graph is represented as $\left|N_{\Gamma }\left(v\right)\right|=d_{v}$. The $N_{\Gamma }\left(v\right)$ denotes the sum of the degrees of the neighbouring vertices of v. Let us define the neighbourhood sum degree-based M-polynomial of Γ,

$$NM\left(\Gamma \right)=\underset{𝒾\leq 𝒿}{\sum }(Number\;of\;all\;edges\;uv\;such\;that\;{ℌ}_{u}=𝒾,\;{ℌ}_{v}=𝒿){𝓇}^{i}{𝓉}^{j}.$$

The degree-based TIs and neighbourhood degree sum-based (ND) TIs are depicted as [10,11]

$$D\left(\Gamma \right)=\underset{uv\epsilon E\left(\Gamma \right)}{\sum }g(\varphi_{u}\varphi_{v})$$

and

$$NM\left(\Gamma \right)=\underset{uv\epsilon E\left(\Gamma \right)}{\sum }g(\omega_{u}\omega_{v})$$

The derivations of NM-Polynomial are listed below in Table 1.

## 3. Neighbourhood Degree Sum-Based Entropy Measures

In his seminal work, Shannon defined entropy as a measure of the unpredictable nature of relevant information or a way of measuring a system’s uncertainty. This paper laid the foundation for modern information theory. The entropy formulae have been used to quantify a network’s structural informativeness [12]. Though information theory was initially used exclusively in electrical engineering and linguistics, its versatile nature found applications in life sciences like chemistry and biology [13] and in graph theory for chemical networks. The notion of graph entropy was proposed to quantify the topological information of chemical networks and graphs.

Rashevsky [14] developed the concept of graph entropy depending on the vertex orbits. The graph entropy measures enable mathematicians to relate graph components such as edges and vertices with probability distributions, categorised as intrinsic and extrinsic measures. Graph entropies have wide-ranging applications in many fields, including chemistry, ecology, sociology, and biology [15,16]. Dehmer introduced graph entropies that captured structural information based on information functionals and studied their properties [17,18]. Estrada et al. [19] introduced a physically-sound graph entropy measure and analysed the walk-based graph entropies [20]. The applications of entropy network measures range from quantitatively describing a molecular structure to exploring biological and chemical features of molecular graphs. Entropy measures have several applications in the field of chemical graph theory. They are used to analyse complex networks and their chemical properties.

Let Γ be a graph with vertex $v_{i}$ and $d_{i}$ be the degree of $v_{i}$ for the given edge $u_{i}v_{j}$, then one can define

$$P_{ij}=\frac{w\left(u_{i}v_{j}\right)}{\sum_{j=1}^{d_{i}}w\left(u_{i}v_{j}\right)}$$

where $w\left(u_{i}v_{j}\right)$ be the weight of the edge $u_{i}v_{j}$ and $w\left(u_{i}v_{j}\right)>0$. The node entropy is defined as

$${\mathrm{ENT}}_{\Gamma }\left(v_{i}\right)=-\sum_{j=1}^{d_{i}}P_{ij}log\left(P_{ij}\right)$$

For an edge-weighted graph Γ=(V,E,w), the entropy measure of *Γ* is defined as [21]

$${\mathrm{ENT}}_{\Gamma }\left(\Gamma ,w\right)=-\sum_{uv\in E(\Gamma )}P_{uv}logP_{uv}$$

where

$$P_{uv}=\frac{w(uv)}{\sum_{uv\in E(\Gamma )}w\left(uv\right)}$$

$${\mathrm{ENT}}_{\chi }\left(\Gamma \right)=-\sum_{uv\in E(\Gamma )}P_{u,v}logP_{u,v}$$

$$=-\sum_{uv\in E(\Gamma )}\frac{F\left(d_{u},d_{v}\right)}{\chi (\Gamma )}log\frac{F\left(d_{u},d_{v}\right)}{\chi (\Gamma )}$$

$$=-\frac{1}{\chi (\Gamma )}\sum_{uv\in E(\Gamma )}F\left(d_{u},d_{v}\right)log\frac{F\left(d_{u},d_{v}\right)}{\chi (\Gamma )}$$

$$=-\frac{1}{\chi (\Gamma )}\sum_{uv\in E(\Gamma )}F\left(d_{u},d_{v}\right)\left(logF\left(d_{u},d_{v}\right)\right)-\left(log\chi \left(\Gamma \right)\right)$$

$$=log\chi \left(\Gamma \right)-\frac{1}{\chi (\Gamma )}\sum_{uv\in E(\Gamma )}F\left(d_{u},d_{v}\right)logF\left(d_{u},d_{v}\right)$$

where TI(Γ)=χ(Γ).

## 4. Computing the Neighbourhood Sum Degree-Based M-polynomial for β-Graphene

In this section, the proper analytical expressions of neighbourhood degree sum-based indices and entropy measures are computed using the M-polynomial for β-Graphene. Graphene is a carbon allotrope, a two-dimensional hexagonal network in which the carbon atoms form vertices with sp^2^ hybridisation. Graphene has many exceptional properties, including mechanical strength, optical transparency, and electric and thermal conductivity. Furthermore, the one-atomic layer structure of graphene makes it ultralight and super thin. Graphene has a thickness of about 0.35 nm, which is approximately 1/200,000th of the thickness of human hair. However, the closely arranged carbon atoms and the sp^2^ orbital hybridisation provide exceptional stability to the graphene structure. Thus, graphene shows extraordinary transparency of 97.7 percent, which means that it only absorbs 2.3 percent of visible light [22].

Due to its high conductivity, graphene can provide a possible alternative to many common substances used as membranes, including indium tin oxide (ITO) and fluorine-doped tin oxide (FTO). The use of graphene for these applications could address the issues of limited indium resources, pollution, and fragility. A membrane with graphene as the primary component could be used as a window barrier in dye-sensitised LEDs and solar cells. It is also possible for graphene to also exist as a nanoribbon in which a lateral charge movement causes an energy barrier to form close to the central point. A reduction in the thickness of the nanoribbon raises this energy barrier. [23]. Hence, by carefully adjusting the width of the graphene nanoribbon, the energy barrier can be accurately regulated, which is a promising advantage for graphene-based electronic devices. Graphene can also be used in the partial detection of external magnetic fields, electric fields, and deformations due to it being a low-noise electrical substance [24].

**Figure 1:**
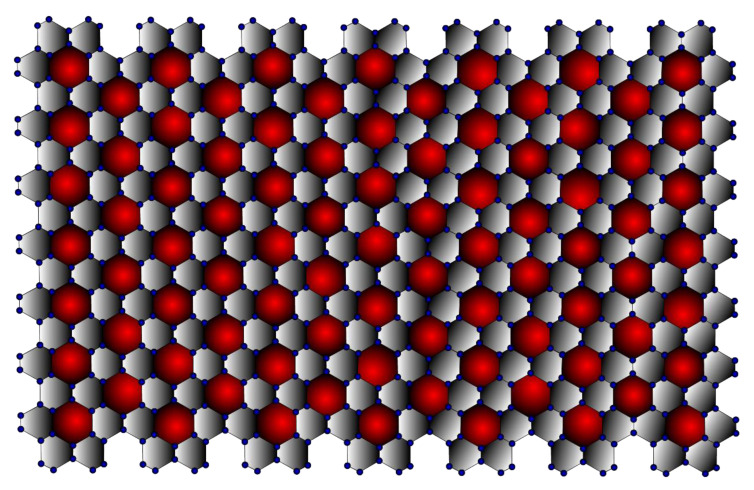
β-Graphene (7,7).

In terms of its structure, graphene can be considered the basic unit of graphite, fullerene [25], carbon nanotube [26], graphyne [27], and other related materials such as amorphous carbon, carbon fibre, charcoal [28], as well as aromatic molecules such as polycyclic aromatic hydrocarbons. As they all have the same structure, they all have some properties in common, even though their different sizes and shapes make them very different. Thus, the structural study of graphene helps to understand the above-listed materials. It can be observed from Figure 1 that the (7,7) β-Graphene contains seven graphene layers stacked next to each other. In three dimensions, the (7,7) β-Graphene contains seven layers stacked on top of each other. Thus, it can be inferred that the (m,n) β-Graphene consists of vertex set V(Γ)=12mn+2m+10n and edge set E(Γ)=18mn+m+11n.

**Theorem** **1.** *If*Γ*is a β-Graphene system, then*NM-*polynomial of*Γ*is given as follows*

$$\begin{array}{l} NM\left(\Gamma ;𝓇,𝓉\right)=\left(4m+2n\right){𝓇}^{5}{𝓉}^{5}+\left(4m+8\right){𝓇}^{5}{𝓉}^{7}+\left(4m+4n-8\right){𝓇}^{5}{𝓉}^{8}+\left(2m+4\right){𝓇}^{7}{𝓉}^{9}\\ \;\;\;\;\;\;\;\;+\left(2n-2\right){𝓇}^{8}{𝓉}^{8}+\left(8m+4n-12\right){𝓇}^{8}{𝓉}^{9}+\left(18mn-21m-n+10\right){𝓇}^{9}{𝓉}^{9} \end{array}$$

**Theorem** **2.** *If*Γ*is a β-Graphene system, then*NM-*polynomial of*Γ*is given as follows*

1. $NMM_{1}\left(\Gamma \right)=324mn-70m+154n$,
2. $NMM_{2}\left(\Gamma \right)=1458mn-599m+545n+30$,
3. $NMM_{2}^{m}\left(\Gamma \right)=\left(\frac{2437}{9450}\right)m+\left(\frac{16489}{64800}\right)n+\left(\frac{2}{9}\right)mn+\frac{1597}{90720}$,
4. $\begin{array}{c} NMR_{\alpha }\left(\Gamma \right)=25^{\varphi }\left(4m+2n\right)+35^{\varphi }\left(4m+8\right)+40^{\varphi }\left(4m+4n-8\right)+63^{\varphi }\left(2m+4\right)+64^{\varphi }\left(2n-2\right)\\ +72^{\varphi }\left(8m+4n-12\right)+81^{\varphi }\left(18mn-21m-n+10\right) \end{array}$,
5. $\begin{array}{c} NMRR_{\alpha }\left(\Gamma \right)=1/25^{\varphi }\left(4m+2n\right)+1/35^{\varphi }\left(4m+8\right)+1/40^{\varphi }\left(4m+4n-8\right)+1/63^{\varphi }\left(2m+4\right)\\ +1/64^{\varphi }\left(2n-2\right)+1/72^{\varphi }\left(8m+4n-12\right)+1/81^{\varphi }\left(18mn-21m-n+10\right) \end{array}$,
6. $NMSDD\left(\Gamma \right)=\left(\frac{151}{42}\right)m+\left(\frac{1033}{45}\right)n+36mn-\frac{503}{630}$,
7. $NMH\left(\Gamma \right)=\left(\frac{12463}{13260}\right)m+\left(\frac{64637}{39780}\right)n+\frac{413}{7956}+2mn$,
8. $NMI\left(\Gamma \right)=\left(\frac{16685}{442}\right)n+81mn-\left(\frac{99547}{5304}\right)m+\frac{1709}{2652}$,
9. $NMA\left(\Gamma \right)=\left(\frac{176959019139103}{233744896000}\right)n+\left(\frac{4782969}{2048}\right)mn-\left(\frac{798535392483}{681472000}\right)m+\frac{11934914516533}{116872448000}$
10. $\begin{array}{c} \mathrm{NMABC}\left(\Gamma \right)=\left(\frac{34}{15}\right)\sqrt{2}m+\left(\frac{4}{5}\right)\sqrt{2}n+\left(\frac{4}{7}\right)\sqrt{2}\sqrt{7}m+\left(\frac{25}{28}\right)\sqrt{2}\sqrt{7}+\left(\frac{1}{5}\right)\sqrt{11}\sqrt{2}\sqrt{5}m+\left(\frac{1}{5}\right)\sqrt{11}\sqrt{2}\sqrt{5}n\\ -\left(\frac{2}{5}\right)\sqrt{11}\sqrt{2}\sqrt{5}+\left(\frac{4}{3}\right)\sqrt{2}+\left(\frac{1}{4}\right)\sqrt{2}\sqrt{7}n+\left(\frac{2}{3}\right)\sqrt{5}\sqrt{3}\sqrt{2}m+\left(1/3\right)\sqrt{5}\sqrt{3}\sqrt{2}n-\sqrt{5}\sqrt{3}\sqrt{2}\\ +8mn-\left(\frac{28}{3}\right)m-\left(\frac{4}{9}\right)n+\frac{40}{9} \end{array}$
11. $\begin{array}{c} NMGA\left(\Gamma \right)=3n+\left(\frac{2}{3}\right)\sqrt{7}\sqrt{5}m+\left(\frac{4}{3}\right)\sqrt{7}\sqrt{5}+\left(\frac{16}{13}\right)\sqrt{2}\sqrt{5}m+\left(\frac{16}{13}\right)\sqrt{2}\sqrt{5}n-\left(\frac{32}{13}\right)\sqrt{2}\sqrt{5}+\left(\frac{3}{4}\right)\sqrt{7}m\\ +\left(\frac{3}{2}\right)\sqrt{7}+8+\left(\frac{96}{17}\right)\sqrt{2}m+\left(\frac{48}{17}\right)\sqrt{2}n-\left(\frac{144}{17}\right)\sqrt{2}+18mn-17m \end{array}$,
12. $NMB_{1}\left(\Gamma \right)=900mn-214m+418n$
13. $NMB_{2}\left(\Gamma \right)=5184mn-2188m+1912n+84$,
14. $NMHB_{1}\left(\Gamma \right)=746496mn-473476m+188680n+4801$

**Proof of Theorem** **2.** *Let* $\begin{array}{l} f\left(𝓇,𝓉\right)=NM\left(\Gamma ;𝓇,𝓉\right)\\ =\left(4m+2n\right){𝓇}^{5}{𝓉}^{5}+\left(4m+8\right){𝓇}^{5}{𝓉}^{7}+\left(4m+4n-8\right){𝓇}^{5}{𝓉}^{8}+\left(2m+4\right){𝓇}^{7}{𝓉}^{9}+\left(2n-2\right){𝓇}^{8}{𝓉}^{8}\\ +\left(8m+4n-12\right){𝓇}^{8}{𝓉}^{9}+\left(18mn-21m-n+10\right){𝓇}^{9}{𝓉}^{9} \end{array}$

$\begin{array}{l} D_{𝓇}\left(\;f\left(𝓇,𝓉\right)\right)=5\left(4m+2n\right){𝓇}^{5}{𝓉}^{5}+5\left(4m+8\right){𝓇}^{5}{𝓉}^{7}+5\left(4m+4n-8\right){𝓇}^{5}{𝓉}^{8}+7\left(2m+4\right){𝓇}^{7}{𝓉}^{9}\\ +8\left(2n-2\right){𝓇}^{8}{𝓉}^{8}+8\left(8m+4n-12\right){𝓇}^{8}{𝓉}^{9}+9\left(18mn-21m-n+10\right){𝓇}^{9}{𝓉}^{9} \end{array}$,

$\begin{array}{l} D_{𝓉}\left(\;f\left(𝓇,𝓉\right)\right)=5\left(4m+2n\right){𝓇}^{5}{𝓉}^{5}+7\left(4m+8\right){𝓇}^{5}{𝓉}^{7}+8\left(4m+4n-8\right){𝓇}^{5}{𝓉}^{8}+9\left(2m+4\right){𝓇}^{7}{𝓉}^{9}\\ +8\left(2n-2\right){𝓇}^{8}{𝓉}^{8}+8\left(8m+4n-12\right){𝓇}^{8}{𝓉}^{9}+9\left(18mn-21m-n+10\right){𝓇}^{9}{𝓉}^{9} \end{array}$,

$\begin{array}{l} D_{𝓇}+D_{𝓉}\left(\;f\left(𝓇,𝓉\right)\right)=10\left(4m+2n\right){𝓇}^{5}{𝓉}^{5}+12\left(4m+8\right){𝓇}^{5}{𝓉}^{7}+13\left(4m+4n-8\right){𝓇}^{5}{𝓉}^{8}+16\left(2m+4\right){𝓇}^{7}{𝓉}^{9}\\ +16\left(2n-2\right){𝓇}^{8}{𝓉}^{8}+17\left(8m+4n-12\right){𝓇}^{8}{𝓉}^{9}+18\left(18mn-21m-n+10\right){𝓇}^{9}{𝓉}^{9} \end{array}$,

$\begin{array}{l} D_{𝓇}D_{𝓉}\left(\;f\left(𝓇,𝓉\right)\right)=25\left(4m+2n\right){𝓇}^{5}{𝓉}^{5}+35\left(4m+8\right){𝓇}^{5}{𝓉}^{7}+40\left(4m+4n-8\right){𝓇}^{5}{𝓉}^{8}+63\left(2m+4\right){𝓇}^{7}{𝓉}^{9}\\ +64\left(2n-2\right){𝓇}^{8}{𝓉}^{8}+72\left(8m+4n-12\right){𝓇}^{8}{𝓉}^{9}+81\left(18mn-21m-n+10\right){𝓇}^{9}{𝓉}^{9} \end{array}$,

$\begin{array}{l} S_{𝓇}\left(\;f\left(𝓇,𝓉\right)\right)=\frac{1}{5}\left(4m+2n\right){𝓇}^{5}{𝓉}^{5}+\frac{1}{5}\left(4m+8\right){𝓇}^{5}{𝓉}^{7}+\frac{1}{5}\left(4m+4n-8\right){𝓇}^{5}{𝓉}^{8}+\frac{1}{7}\left(2m+4\right){𝓇}^{7}{𝓉}^{9}\\ +\frac{1}{8}\left(2n-2\right){𝓇}^{8}{𝓉}^{8}+\frac{1}{8}\left(8m+4n-12\right){𝓇}^{8}{𝓉}^{9}+\frac{1}{9}\left(18mn-21m-n+10\right){𝓇}^{9}{𝓉}^{9} \end{array}$,

$\begin{array}{l} S_{𝓉}\left(\;f\left(𝓇,𝓉\right)\right)=\frac{1}{5}\left(4m+2n\right){𝓇}^{5}{𝓉}^{5}+\frac{1}{7}\left(4m+8\right){𝓇}^{5}{𝓉}^{7}+\frac{1}{8}\left(4m+4n-8\right){𝓇}^{5}{𝓉}^{8}+\frac{1}{9}\left(2m+4\right){𝓇}^{7}{𝓉}^{9}\\ +\frac{1}{8}\left(2n-2\right){𝓇}^{8}{𝓉}^{8}+\frac{1}{9}\left(8m+4n-12\right){𝓇}^{8}{𝓉}^{9}+\frac{1}{9}\left(18mn-21m-n+10\right){𝓇}^{9}{𝓉}^{9} \end{array}$,

$\begin{array}{l} S_{𝓉}S_{𝓇}\left(f\left(𝓇,𝓉\right)\right)=\frac{1}{25}\left(4m+2n\right){𝓇}^{5}{𝓉}^{5}+\frac{1}{35}\left(4m+8\right){𝓇}^{5}{𝓉}^{7}+\frac{1}{40}\left(4m+4n-8\right){𝓇}^{5}{𝓉}^{8}+\frac{1}{63}\left(2m+4\right){𝓇}^{7}{𝓉}^{9}\\ +\frac{1}{64}\left(2n-2\right){𝓇}^{8}{𝓉}^{8}+\frac{1}{72}\left(8m+4n-12\right){𝓇}^{8}{𝓉}^{9}+\frac{1}{81}\left(18mn-21m-n+10\right){𝓇}^{9}{𝓉}^{9} \end{array}$,

$\begin{array}{l} D_{𝓇}^{\varphi }D_{𝓉}^{\varphi }\left(\;f\left(𝓇,𝓉\right)\right)=25^{\varphi }\left(4m+2n\right){𝓇}^{5}{𝓉}^{5}+35^{\varphi }\left(4m+8\right){𝓇}^{5}{𝓉}^{7}+40^{\varphi }\left(4m+4n-8\right){𝓇}^{5}{𝓉}^{8}+63^{\varphi }\left(2m+4\right){𝓇}^{7}{𝓉}^{9}\\ +64^{\varphi }\left(2n-2\right){𝓇}^{8}{𝓉}^{8}+72^{\varphi }\left(8m+4n-12\right){𝓇}^{8}{𝓉}^{9}+81^{\varphi }\left(18mn-21m-n+10\right){𝓇}^{9}{𝓉}^{9} \end{array}$,

$\begin{array}{l} S_{𝓇}^{\varphi }S_{𝓉}^{\varphi }\left(f\left(𝓇,𝓉\right)\right)=\frac{1}{25^{\varphi }}\left(4m+2n\right){𝓇}^{5}{𝓉}^{5}+\frac{1}{35^{\varphi }}\left(4m+8\right){𝓇}^{5}{𝓉}^{7}+\frac{1}{40^{\varphi }}\left(4m+4n-8\right){𝓇}^{5}{𝓉}^{8}+\frac{1}{63^{\varphi }}\left(2m+4\right){𝓇}^{7}{𝓉}^{9}\\ +\frac{1}{64^{\varphi }}\left(2n-2\right){𝓇}^{8}{𝓉}^{8}+\frac{1}{72^{\varphi }}\left(8m+4n-12\right){𝓇}^{8}{𝓉}^{9}+\frac{1}{81^{\varphi }}\left(18mn-21m-n+10\right){𝓇}^{9}{𝓉}^{9} \end{array}$,

$\begin{array}{l} S_{𝓉}D_{𝓇}\left(\;f\left(𝓇,𝓉\right)\right)=1\left(4m+2n\right){𝓇}^{5}{𝓉}^{5}+\frac{5}{7}\left(4m+8\right){𝓇}^{5}{𝓉}^{7}+\frac{5}{8}\left(4m+4n-8\right){𝓇}^{5}{𝓉}^{8}+\frac{7}{9}\left(2m+4\right){𝓇}^{7}{𝓉}^{9}\\ +1\left(2n-2\right){𝓇}^{8}{𝓉}^{8}+\frac{8}{9}\left(8m+4n-12\right){𝓇}^{8}{𝓉}^{9}+1\left(18mn-21m-n+10\right){𝓇}^{9}{𝓉}^{9} \end{array}$,

$\begin{array}{l} S_{𝓇}D_{𝓉}\left(\;f\left(𝓇,𝓉\right)\right)=1\left(4m+2n\right){𝓇}^{5}{𝓉}^{5}+\frac{7}{5}\left(4m+8\right){𝓇}^{5}{𝓉}^{7}+\frac{8}{5}\left(4m+4n-8\right){𝓇}^{5}{𝓉}^{8}+\frac{9}{7}\left(2m+4\right){𝓇}^{7}{𝓉}^{9}\\ +1\left(2n-2\right){𝓇}^{8}{𝓉}^{8}+\frac{9}{8}\left(8m+4n-12\right){𝓇}^{8}{𝓉}^{9}+1\left(18mn-21m-n+10\right){𝓇}^{9}{𝓉}^{9} \end{array}$,

$\begin{array}{l} J\left(\;f\left(𝓇,𝓉\right)\right)=f\left(𝓇,𝓇\right)=\left(4m+2n\right){𝓇}^{10}+\left(4m+8\right){𝓇}^{12}+\left(4m+4n-8\right){𝓇}^{13}+\left(2m+4\right){𝓇}^{16}\\ +\left(2n-2\right){𝓇}^{16}+\left(8m+4n-12\right){𝓇}^{17}+9\left(18mn-21m-n+10\right){𝓇}^{18} \end{array}$,

$\begin{array}{l} S_{𝓇}J\;f\left(𝓇,𝓉\right)=\frac{1}{10}\left(4m+2n\right){𝓇}^{10}+\frac{1}{12}\left(4m+8\right){𝓇}^{12}+\frac{1}{13}\left(4m+4n-8\right){𝓇}^{13}+\frac{1}{16}\left(2m+4\right){𝓇}^{16}\\ +\frac{1}{16}\left(2n-2\right){𝓇}^{16}+\frac{1}{17}\left(8m+4n-12\right){𝓇}^{17}+\frac{1}{18}\left(18mn-21m-n+10\right){𝓇}^{18} \end{array}$,

$\begin{array}{l} S_{𝓇}J\;D_{𝓇}D_{𝓉}f\left(𝓇,𝓉\right)=\frac{25}{10}\left(4m+2n\right){𝓇}^{10}+\frac{35}{12}\left(4m+8\right){𝓇}^{12}+\frac{40}{13}\left(4m+4n-8\right){𝓇}^{13}+\frac{63}{16}\left(2m+4\right){𝓇}^{16}\\ +\frac{64}{16}\left(2n-2\right){𝓇}^{16}+\frac{72}{17}\left(8m+4n-12\right){𝓇}^{17}+\frac{81}{18}\left(18mn-21m-n+10\right){𝓇}^{18} \end{array}$,

$\begin{array}{l} S_{𝓇}^{3}Q_{-2}J\;D_{𝓇}^{3}D_{𝓉}^{3}f\left(𝓇,𝓉\right)={\left(\frac{25}{8}\right)}^{3}\left(4m+2n\right){𝓇}^{8}+{\left(\frac{7}{2}\right)}^{3}\left(4m+8\right){𝓇}^{10}+{\left(\frac{40}{11}\right)}^{3}\left(4m+4n-8\right){𝓇}^{11}\\ +{\left(\frac{9}{2}\right)}^{3}\left(2m+4\right){𝓇}^{14}+{\left(\frac{32}{7}\right)}^{3}\left(2n-2\right){𝓇}^{14}+{\left(\frac{24}{5}\right)}^{3}\left(8m+4n-12\right){𝓇}^{15}+{\left(\frac{81}{16}\right)}^{3}\left(18mn-21m-n+10\right){𝓇}^{16} \end{array}$,

$\begin{array}{l} D_{𝓇}^{\frac{1}{2}}Q_{-2}J\;S_{𝓇}^{\frac{1}{2}}S_{𝓉}^{\frac{1}{2}}f\left(𝓇,𝓉\right)=\frac{\sqrt{8}}{5}\left(4m+2n\right){𝓇}^{8}+\frac{\sqrt{10}}{\sqrt{35}}\left(4m+8\right){𝓇}^{10}+\frac{\sqrt{11}}{\sqrt{40}}\left(4m+4n-8\right){𝓇}^{11}\\ +\frac{\sqrt{14}}{3\sqrt{7}}\left(2m+4\right){𝓇}^{14}+\frac{\sqrt{14}}{8}\left(2n-2\right){𝓇}^{14}+\frac{\sqrt{15}}{3\sqrt{8}}\left(8m+4n-12\right){𝓇}^{15}+\frac{\sqrt{16}}{9}\left(18mn-21m-n+10\right){𝓇}^{16} \end{array}$,

$\begin{array}{l} 2S_{𝓇}JD_{𝓇}^{\frac{1}{2}}D_{t}^{\frac{1}{2}}f\left(𝓇,𝓉\right)=\frac{10}{10}\left(4m+2n\right){𝓇}^{10}+\frac{2\sqrt{35}}{12}\left(4m+8\right){𝓇}^{12}+\frac{2\sqrt{40}}{13}\left(4m+4n-8\right){𝓇}^{13}+\frac{2\sqrt{63}}{16}\left(2m+4\right){𝓇}^{16}\\ +\frac{16}{16}\left(2n-2\right){𝓇}^{16}+\frac{2\sqrt{72}}{17}\left(8m+4n-12\right){𝓇}^{17}+\frac{18}{18}\left(18mn-21m-n+10\right){𝓇}^{18} \end{array}$,

$\begin{array}{l} 2D_{𝓇}Q_{-2}Jf\left(𝓇,𝓉\right)=2\cdot 8\left(4m+2n\right){𝓇}^{8}+2\cdot 10\left(4m+8\right){𝓇}^{10}+2\cdot 11\left(4m+4n-8\right){𝓇}^{11}+2\cdot 14\left(2m+4\right){𝓇}^{14}\\ +2\cdot 14\left(2n-2\right){𝓇}^{14}+2\cdot 15\left(8m+4n-12\right){𝓇}^{15}+2\cdot 16\left(18mn-21m-n+10\right){𝓇}^{16} \end{array}$,

$\begin{array}{l} D_{𝓇}Q_{-2}J\left(D_{𝓇}+D_{𝓉}\right)f\left(𝓇,𝓉\right)=8\cdot 10\left(4m+2n\right){𝓇}^{8}+10\cdot 12\left(4m+8\right){𝓇}^{10}+11\cdot 13\left(4m+4n-8\right){𝓇}^{11}\\ +14\cdot 16\left(2m+4\right){𝓇}^{14}+14\cdot 16\left(2n-2\right){𝓇}^{14}+15\cdot 17\left(8m+4n-12\right){𝓇}^{15}+16\cdot 18\left(18mn-21m-n+10\right){𝓇}^{16} \end{array}$,

$\begin{array}{l} D_{𝓇}^{2}Q_{-2}J\left(D_{𝓇}^{2}+D_{𝓉}^{2}\right)f\left(𝓇,𝓉\right)=64\cdot 50\left(4m+2n\right){𝓇}^{8}+100\cdot 74\left(4m+8\right){𝓇}^{10}+121\cdot 89\left(4m+4n-8\right){𝓇}^{11}\\ +196\cdot 130\left(2m+4\right){𝓇}^{14}+196\cdot 128\left(2n-2\right){𝓇}^{14}+225\cdot 145\left(8m+4n-12\right){𝓇}^{15}+256\cdot 162\left(18mn-21m-n+10\right){𝓇}^{16} \end{array}$.

The above results are obtained by using the conditions of M-Polynomial with its derivatives, and the partition Table 2. □

## 5. Neighbourhood Degree Sum-Based Entropy Measures for β-Graphene

**Theorem** **3.** *If*Γ*is a β-Graphene system, then*NM-*entropy measures of*Γ*are given as follows*

1. $\begin{array}{cc} NENT_{NM_{1}}\left(\Gamma \right)= & log(324mn-70m+154n)\\ \; & -\frac{1}{324mn-70m+154n}[\left(4m+2n\right)\left(10\right)log10+\left(4m+8\right)\left(12\right)log12\\ \; & +(4m+4n-8)(13)log13+(2m+4)(16)log16+(2n-2)(16)log16\\ \; & +(8m+4n-12)(17)log17+(18mn-21m-n+10)(18)log18] \end{array}$
  $=log\left(324mn-70m+154n\right)-\left(\frac{-273.7788m+408.7804n+2.8232+936.4896mn}{324mn-70m+154n}\right)$
2. $\begin{array}{cc} NENT_{NM_{2}}\left(\Gamma \right)= & log(1458mn-599m+545n+30)\\ \; & -\frac{1}{1458mn-599m+545n+30}[\left(4m+2n\right)\left(25\right)log25+\left(4m+8\right)\left(35\right)log35\\ \; & +(4m+4n-8)(40)log40+(2m+4)(63)log63+(2n-2)(64)log64\\ \; & +(8m+4n-12)(72)log72+(18mn-21m-n+10)(81)log81] \end{array}$
  $=log\left(1458mn-599m+545n+30\right)-\left(\frac{-3079.6186m+2159.2464n+191.1532+6407.0352mn}{1458mn-599m+545n+30}\right)$
3. $\begin{array}{cc} {\mathrm{NNET}}_{NM_{2}^{m}}\left(\Gamma \right)= & log\left(\left(\frac{2437}{9450}\right)m+\left(\frac{16489}{64800}\right)n+\left(\frac{2}{9}\right)mn+\frac{1597}{90720}\right)\\ \; & -\frac{1}{\left(\frac{2437}{9450}\right)m+\left(\frac{16489}{64800}\right)n+\left(\frac{2}{9}\right)mn+\frac{1597}{90720}}\left[\left(4m+2n\right)\left(\frac{1}{25}\right)log\frac{1}{25}+(4m\right.\\ \; & +8)\left(\frac{1}{35}\right)log\frac{1}{35}+\left(4m+4n-8\right)\left(\frac{1}{40}\right)log\frac{1}{40}+\left(2m+4\right)\left(\frac{1}{63}\right)log\frac{1}{63}+(2n\\ \; & -2)\left(\frac{1}{64}\right)log\frac{1}{64}+\left(8m+4n-12\right)\left(\frac{1}{72}\right)log\frac{1}{72}+(18mn-21m-n\\ \; & \left.+10)\left(\frac{1}{81}\right)log\frac{1}{81}\right] \end{array}$
  $=log\left(\left(\frac{2437}{9450}\right)m+\left(\frac{16489}{64800}\right)n+\left(\frac{2}{9}\right)mn+\frac{1597}{90720}\right)-\left(\frac{-.7576449840m-.9397022176n-0.376835284e-1-.9765333333mn}{\left(\frac{2437}{9450}\right)m+\left(\frac{16489}{64800}\right)n+\left(\frac{2}{9}\right)mn+\frac{1597}{90720}}\right)$
4. $\begin{array}{cc} NENT_{NHM}\left(\Gamma \right)= & log(5832mn-2328m+2220n+84)\\ \; & -\frac{1}{5832mn-2328m+2220n+84}[\left(4m+2n\right)\left(100\right)log100\\ \; & +(4m+8)(144)log144+(4m+4n-8)(169)log169+(2m+4)(256)log256\\ \; & +(2n-2)(256)log256+(8m+4n-12)(289)log289\\ \; & +(18mn-21m-n+10)(324)log324] \end{array}$
  $=log\left(5832mn-2328m+2220n+84\right)-\left(\frac{-15220.2744m+11905.3064n+707.5792+33713.6256mn}{5832mn-2328m+2220n+84}\right)$
5. $\begin{array}{c} NENT_{NA}\left(\Gamma \right)\\ =log\left(\left(\frac{4782969}{2048}\right)mn-\left(\frac{798535392483}{681472000}\right)m+\left(\frac{176959019139103}{233744896000}\right)n+\frac{11934914516533}{116872448000}\right)\\ -\frac{1}{\left(\frac{4782969}{2048}\right)mn-\left(\frac{798535392483}{681472000}\right)m+\left(\frac{176959019139103}{233744896000}\right)n+\frac{11934914516533}{116872448000}}[(4m\\ +2n){\left(\frac{25}{8}\right)}^{3}log{\left(\frac{25}{8}\right)}^{3}+\left(4m+8\right){\left(\frac{35}{10}\right)}^{3}log\frac{35^{3}}{10}+\left(4m+4n-8\right){\left(\frac{40}{11}\right)}^{3}log\frac{40^{3}}{11}\\ +\left(2m+4\right){\left(\frac{63}{14}\right)}^{3}log\frac{63^{3}}{14}+\left(2n-2\right){\left(\frac{64}{14}\right)}^{3}log\frac{64^{3}}{14}+\left(8m+4n-12\right){\left(\frac{72}{15}\right)}^{3}log\frac{72^{3}}{15}\\ \left.+\left(18mn-21m-n+10\right){\left(\frac{81}{16}\right)}^{3}log\frac{81^{3}}{16}\right] \end{array}$
  $=log\left(\left(\frac{4782969}{2048}\right)mn-\left(\frac{798535392483}{681472000}\right)m+\left(\frac{176959019139103}{233744896000}\right)n+\frac{11934914516533}{116872448000}\right)-\frac{-6658.292895m+2328.656416n+829.875339+9964.129511mn}{\left(\frac{4782969}{2048}\right)mn-\left(\frac{798535392483}{681472000}\right)m+\left(\frac{176959019139103}{233744896000}\right)n+\frac{11934914516533}{116872448000}}$
6. $\begin{array}{cc} NENT_{NABC}\left(\Gamma \right)= & log(NABC(\Gamma ))\\ \; & -\frac{1}{NABC(\Gamma )}\left[\left(4m+2n\right)\left(\sqrt{\frac{8}{25}}\right)log\sqrt{\frac{8}{25}}+\left(4m+8\right)\left(\sqrt{\frac{10}{35}}\right)log\sqrt{\frac{10}{35}}\right.\\ \; & +\left(4m+4n-8\right)\left(\sqrt{\frac{11}{40}}\right)log\sqrt{\frac{11}{40}}+\left(2m+4\right)\left(\sqrt{\frac{14}{63}}\right)log\sqrt{\frac{14}{63}}\\ \; & +\left(2n-2\right)\left(\sqrt{\frac{14}{64}}\right)log\sqrt{\frac{14}{64}}+\left(8m+4n-12\right)\left(\sqrt{\frac{15}{72}}\right)log\sqrt{\frac{15}{72}}\\ \; & \left.+\left(18mn-21m-n+10\right)\left(\sqrt{\frac{16}{81}}\right)log\sqrt{\frac{16}{81}}\right] \end{array}$
7. $\begin{array}{cc} NENT_{NGA}\left(\Gamma \right)= & log(NGA(\Gamma ))\\ \; & -\frac{1}{NGA(\Gamma )}\left[\left(4m+2n\right)\left(1\right)log1+\left(4m+8\right)\left(\frac{2\sqrt{35}}{12}\right)log\frac{2\sqrt{35}}{12}\right.\\ \; & +\left(4m+4n-8\right)\left(\frac{2\sqrt{40}}{13}\right)log\frac{2\sqrt{40}}{13}+\left(2m+4\right)\left(\frac{2\sqrt{63}}{16}\right)log\frac{2\sqrt{63}}{16}+\left(2n-2\right)\left(1\right)log1\\ \; & \left.+\left(8m+4n-12\right)\left(\frac{2\sqrt{72}}{17}\right)log\frac{2\sqrt{72}}{17}+\left(18mn-21m-n+10\right)\left(1\right)log1\right] \end{array}$
8. $\begin{array}{cc} {\mathrm{NENT}}_{NAG_{1}}\left(\Gamma \right)= & log\left(AG_{1}\left(\Gamma \right)\right)\\ \; & -\frac{1}{AG_{1}\left(\Gamma \right)}\left[\left(4m+2n\right)\left(1\right)log1+\left(4m+8\right)\left(\frac{12}{2\sqrt{35}}\right)log\frac{12}{2\sqrt{35}}\right.\\ \; & +\left(4m+4n-8\right)\left(\frac{13}{2\sqrt{40}}\right)log\frac{13}{2\sqrt{40}}+\left(2m+4\right)\left(\frac{16}{2\sqrt{63}}\right)log\frac{16}{2\sqrt{63}}+\left(2n-2\right)\left(1\right)log1\\ \; & \left.+\left(8m+4n-12\right)\left(\frac{17}{2\sqrt{72}}\right)log\frac{17}{2\sqrt{72}}+\left(18mn-21m-n+10\right)\left(1\right)log1\right] \end{array}$
9. $\begin{array}{cc} NENT_{NF_{1}}\left(\Gamma \right)= & log(2916mn-1130m+1130n+24)\\ \; & -\frac{1}{2916mn-1130m+1130n+24}[\left(4m+2n\right)\left(50\right)log50+\left(4m+8\right)\left(74\right)log74\\ \; & +(4m+4n-8)(89)log89+(2m+4)(130)log130+(2n-2)(128)log128\\ \; & +(8m+4n-12)(145)log145+(18mn-21m-n+10)(162)log162] \end{array}$
  $=log(2916mn-1130m+1130n+24)-\left(\frac{-6615.1380m+5293.5484n+223.5860+14835.4416mn}{2916mn-1130m+1130n+24}\right)$
10. $\begin{array}{cc} NENT_{NF_{2}}\left(\Gamma \right)= & log(118098mn-74571m+30017n+8086)\\ \; & -\frac{1}{118098mn-74571m+30017n+8086}[\left(4m+2n\right)\left(625\right)log625\\ \; & +(4m+8)(1225)log1225+(4m+4n-8)(1600)log1600\\ \; & +(2m+4)(3969)log3969+(2n-2)(4096)log4096\\ \; & +(8m+4n-12)(5184)log5184+(18mn-21m-n+10)(6561)log6561] \end{array}$
  $=log\left(118098mn-74571m+30017n+8086\right)-\left(\frac{-6.922733326\times 10^{5}m+2.431043232\times 10^{5}n+83203.5944+1.037939702\times 10^{6}mn}{118098mn-74571m+30017n+8086}\right)$
11. $\begin{array}{c} NENT_{N\chi }\left(\Gamma \right)=log\left(N\chi \left(\Gamma \right)\right)-\frac{1}{N\chi (\Gamma )}\left[\left(4m+2n\right)\left(\frac{1}{\sqrt{10}}\right)log\frac{1}{\sqrt{10}}+\left(4m+8\right)\left(\frac{1}{\sqrt{12}}\right)log\frac{1}{\sqrt{12}}+\right.\\ \left(4m+4n-8\right)\left(\frac{1}{\sqrt{13}}\right)log\frac{1}{\sqrt{13}}+\left(2m+4\right)\left(\frac{1}{\sqrt{16}}\right)log\frac{1}{\sqrt{16}}+\left(2n-2\right)\left(\frac{1}{\sqrt{16}}\right)log\frac{1}{\sqrt{16}}+(8m+\\ \left.4n-12)\left(\frac{1}{\sqrt{17}}\right)log\frac{1}{\sqrt{17}}+\left(18mn-21m-n+10\right)\left(\frac{1}{\sqrt{18}}\right)log\frac{1}{\sqrt{18}}\right] \end{array}$
12. $\begin{array}{c} NENT_{N\mathrm{ReZ}\Gamma_{1}}\left(\Gamma \right)=log\left(\left(\frac{146}{35}\right)m+\left(\frac{274}{35}\right)n+\left(\frac{516}{35}\right)mn\right)-\frac{1}{\left(\frac{146}{35}\right)m+\left(\frac{274}{35}\right)n+\left(\frac{516}{35}\right)mn}[(4m+\\ 2n)\left(\frac{10}{25}\right)log\frac{10}{25}+\left(4m+8\right)\left(\frac{12}{35}\right)log\frac{12}{35}+\left(4m+4n-8\right)\left(\frac{13}{40}\right)log\frac{13}{40}+(2m+\\ 4)\left(\frac{16}{63}\right)log\frac{16}{63}+\left(2n-2\right)\left(\frac{16}{64}\right)log\frac{16}{64}+\left(8m+4n-12\right)\left(\frac{17}{72}\right)log\frac{17}{72}+(18mn-21m-\\ \left.n+10)\left(\frac{18}{81}\right)log\frac{18}{81}\right] \end{array}$
  $=log\left(\left(\frac{146}{35}\right)m+\left(\frac{274}{35}\right)n+\left(\frac{516}{35}\right)mn\right)-\left(\frac{-.798715906m-3.916313112n+0.34553969e-1-6.016399999mn}{\left(\frac{146}{35}\right)m+\left(\frac{274}{35}\right)n+\left(\frac{516}{35}\right)mn}\right)$
13. $\begin{array}{cc} NENT_{NReZG_{2}}\left(\Gamma \right)= & log\left(\left(\frac{1309}{18}\right)n+121mn-\left(\frac{451}{18}\right)m\right)\\ \; & -\frac{1}{\left(\frac{1309}{18}\right)n+121mn-\left(\frac{451}{18}\right)m}\left[\left(4m+2n\right)\left(\frac{25}{10}\right)log\frac{25}{10}+\left(4m+8\right)\left(\frac{35}{12}\right)log\frac{35}{12}\right.\\ \; & +\left(4m+4n-8\right)\left(\frac{40}{13}\right)log\frac{40}{13}+\left(2m+4\right)\left(\frac{63}{16}\right)log\frac{63}{16}+\left(2n-2\right)\left(\frac{64}{16}\right)log\frac{64}{16}\\ \; & \left.+\left(8m+4n-12\right)\left(\frac{72}{17}\right)log\frac{72}{17}+\left(18mn-21m-n+10\right)\left(\frac{81}{18}\right)log\frac{81}{18}\right] \end{array}$
  $=ln\left(\left(\frac{1309}{18}\right)n+121mn-\left(\frac{451}{18}\right)m\right)-\left(\frac{-46.95207065m+47.19060362n+2.12647952+121.8321000mn}{\left(\frac{1309}{18}\right)n+121mn-\left(\frac{451}{18}\right)m}\right)$
14. $\begin{array}{cc} NENT_{NReZG_{3}}\left(\Gamma \right) & =log(26244mn-14050m+8066n+1076)\\ \; & -\frac{1}{26244mn-14050m+8066n+1076}[\left(4m+2n\right)\left(250\right)log250\\ \; & +(4m+8)(420)log420+(4m+4n-8)(520)log520+(2m+4)(1008)log1008\\ \; & +(2n-2)(1024)log1024+(8m+4n-12)(1224)log1224\\ \; & +(18mn-21m-n+10)(1458)log1458] \end{array}$
  $=log\left(26244mn-14050m+8066n+1076\right)-\left(\frac{-1.108067064\times 10^{5}m+54153.1980n+9750.1632+1.911822912\times 10^{5}mn}{26244mn-14050m+8066n+1076}\right)$

where $NABC\left(\Gamma \right),NMGA\left(\Gamma \right),NAG_{1}\left(\Gamma \right)$ and Nχ(Γ) can be represented as $\begin{array}{c} NABC\left(\Gamma \right)=\left(\frac{34}{15}\right)\sqrt{2}m+\left(\frac{4}{5}\right)\sqrt{2}n+\left(\frac{4}{7}\right)\sqrt{2}\sqrt{7}m+\left(\frac{25}{28}\right)\sqrt{2}\;\sqrt{7}+\left(\frac{1}{5}\right)\sqrt{2}\;\sqrt{5}\;\sqrt{11}m+\left(\frac{1}{5}\right)\sqrt{2}\sqrt{5}\;\sqrt{11}n\\ -\left(\frac{2}{5}\right)\sqrt{2}\sqrt{5}\;\sqrt{11}+\left(\frac{4}{3}\right)\sqrt{2}+\left(\frac{1}{4}\right)\sqrt{2}\sqrt{7}n+\left(\frac{2}{3}\right)\sqrt{2}\sqrt{5}\sqrt{3}m+\left(\frac{1}{3}\right)\sqrt{2}\sqrt{5}\;\sqrt{3}n-s\sqrt{2}\sqrt{5}\sqrt{3}+8mn-\left(\frac{28}{3}\right)m-\left(\frac{4}{9}\right)n+\frac{40}{9} \end{array}$, $NMGA\left(\Gamma \right)=18mn-17m+3n+\left(\frac{2}{3}\right)\sqrt{7}\sqrt{5}m+\left(\frac{4}{3}\right)\sqrt{7}\sqrt{5}+\left(\frac{16}{13}\right)\sqrt{2}\sqrt{5}m+\left(\frac{16}{13}\right)\sqrt{2}\sqrt{5}n-\left(\frac{32}{13}\right)\sqrt{2}\sqrt{5}+\left(\frac{3}{4}\right)\sqrt{7}m+\left(\frac{3}{2}\right)\sqrt{7}+8+\left(\frac{96}{17}\right)\sqrt{2}m+\left(\frac{48}{17}\right)\sqrt{2}\;n-\left(\frac{144}{17}\right)\sqrt{2}$, $NMAG_{1}=18mn-17m+3n+\left(\frac{24}{35}\right)\sqrt{7}\sqrt{5}m+\left(\frac{48}{35}\right)\sqrt{7}\sqrt{5}+\left(\frac{13}{10}\right)\sqrt{2}\sqrt{5}m+\left(\frac{13}{10}\right)\sqrt{2}\sqrt{5}n-\left(\frac{13}{5}\right)\sqrt{2}\sqrt{5}+\left(\frac{16}{21}\right)\sqrt{7}m+\left(\frac{32}{21}\right)\sqrt{7}+8+\left(\frac{17}{3}\right)\sqrt{2}m+\left(\frac{17}{6}\right)\sqrt{2}n-\left(\frac{17}{2}\right)\sqrt{2}$ and $\begin{array}{c} NM\chi \left(\Gamma \right)=\left(\frac{2}{5}\right)\sqrt{2}\sqrt{5}m+\left(\frac{1}{5}\right)\sqrt{2}\sqrt{5}n+\left(\frac{2}{3}\right)\sqrt{3}m+\left(\frac{4}{3}\right)\sqrt{3}+\left(\frac{4}{13}\right)\sqrt{13}m+\left(\frac{4}{13}\right)\sqrt{13}n-\left(\frac{8}{13}\right)\sqrt{13}+\left(\frac{1}{2}\right)m+\frac{1}{2}+\left(\frac{1}{2}\right)n\\ +\left(\frac{8}{17}\right)\sqrt{17}m+\left(\frac{4}{17}\right)\sqrt{17}n-\left(\frac{12}{17}\right)\sqrt{17}+3\sqrt{2}mn-\left(\frac{7}{2}\right)\sqrt{2}m-\left(\frac{1}{6}\right)\sqrt{2}n+\left(\frac{5}{3}\right)\sqrt{2} \end{array}$.

## 6. Comparative Analysis for β-Graphene

**Figure 2:**
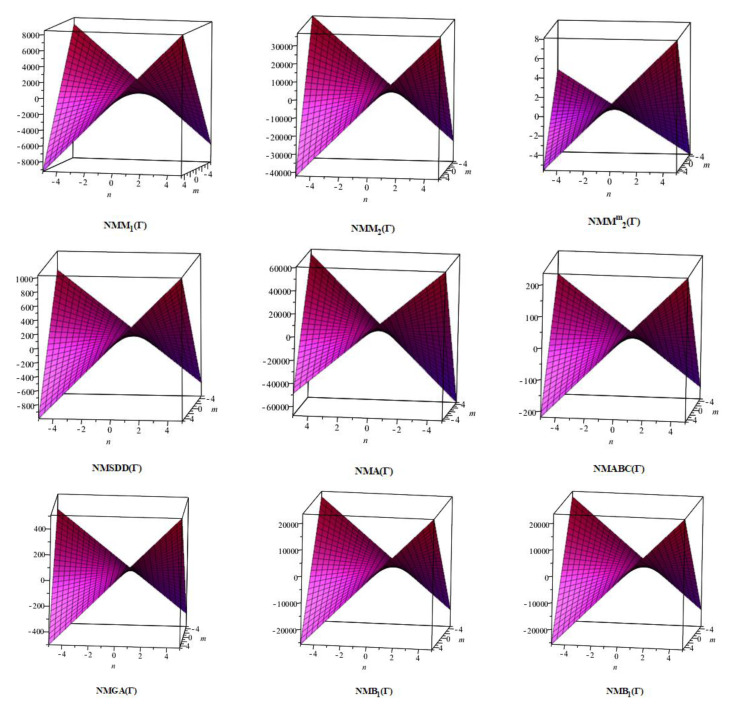
3D plots for Theorem 2.

**Figure 3:**
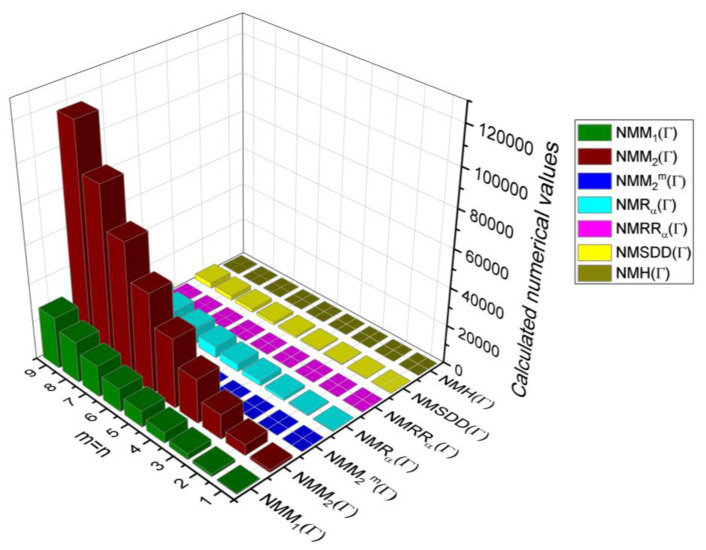
3D Plots for Table 3.

**Figure 4:**
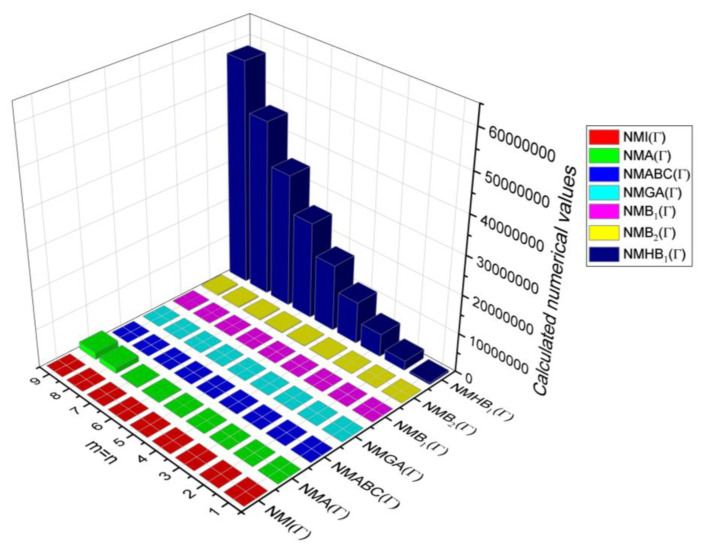
3D Plots for Table 4.

**Figure 5:**
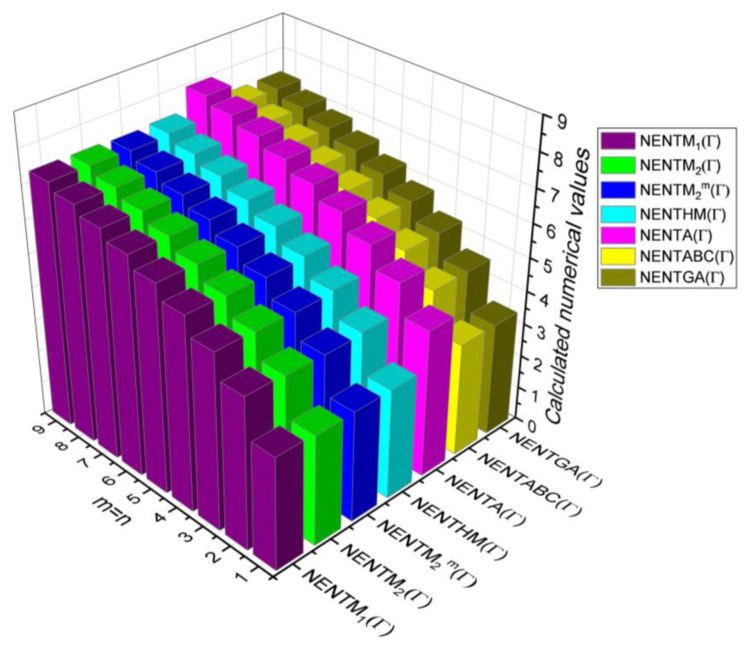
3D Plots for Table 5.

**Figure 6:**
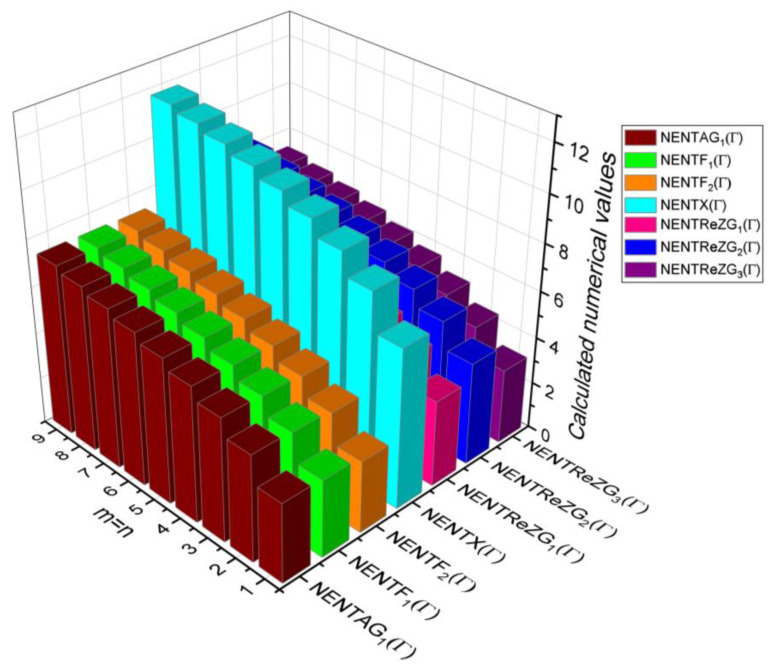
3D Plots for Table 6.

In this section, the analytical expressions of the neighbourhood degree sum-based indices derived from Theorem 2 and Theorem 3 are represented as 3D plots. These plots help the reader visually interpret and understand the behaviour of the indices with respect to the variables that define the molecular structure. In addition, the results of Theorem 2 have also been represented as comparison plots where all the neighbourhood degree sum-based indices are plotted in the same graph against the same structural variables. These comparison plots provide a graphical representation of how the indices vary with respect to each other and the molecular structure. These plots are presented in Figure 2 and Figure 3. The numerical values of the indices are computed and listed in Table 3, Table 4, Table 5 and Table 6. The numerical values are represented as 3D plots in Figure 3, Figure 4, Figure 5, and Figure 6 respectively to provide a visual understanding to the readers.

## 7. Neighbourhood Degree Sum-Based M-Polynomial for β-Graphyne

Graphyne is a type of graphene in which the hexagons are connected by acetylenic bonds, as shown in Figure 1. Graphyne is especially intriguing because its distinct electronic structure sets it apart from other commonly available carbon allotropes, such as diamond and graphite. Furthermore, adding transition metals to graphyne sheets significantly affects the material’s general behaviour [29]. For example, the adsorption of chromium and iron transforms graphyne from a semiconductor into a spin-polarised metal. Other transition metal adsorption can produce a spin-polarised half semiconductor or a narrow gap semiconductor.

Computational studies demonstrate that the band gap of graphyne is mechanically adjustable, which facilitates the fabrication of transistors with various characteristics that are directly linked to the band gap. It also allows for the determination of these properties much later in the manufacturing process when compared to conventional semiconductors, where the properties are controlled based on doping [30,31,32].

**Figure 7:**
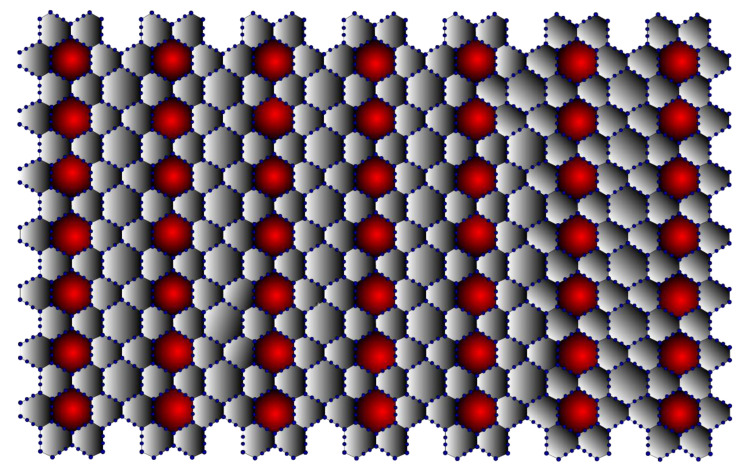
β-Graphyne (7,7).

Moreover, research has revealed that graphyne possesses peculiar optical properties. Although graphyne has not yet been successfully synthesised, its properties can be used in a variety of applications, including anisotropic conductors, nanofillers, desalinators, semiconductor metal hybrids, transistors, and sensors. The mechanical properties of graphyne enable its use as a nanofiller in composite materials. A sizeable elastic strain region of graphyne also makes it possible to drastically alter the material and revert it to its original shape without lasting damage [32]. This property allows for resilient electromechanical coupling, which is advantageous for many applications, including temperature sensing. The cardinality of β-Graphynes is V(Γ)=36mn+2m+24n and E(Γ)=42mn+m+23n. It is represented in Figure 7. The edge partitions for the β-Graphynes are presented in Table 7.

**Theorem** **4.** *If*Γ*is a β- graphyne system, then*NM-*polynomial of*Γ*is given as follows*

$$\begin{array}{l} NM\left(\Gamma ;𝓇,𝓉\right)=\left(12m+6n\right){𝓇}^{4}{𝓉}^{4}+\left(8m+4n\right){𝓇}^{4}{𝓉}^{5}\\ \;\;\;\;\;\;\;\;\;\;\;\;\;\;\;\;\;\;\;\;\;\;\;\;\;\;\;\;\;+\left(12mn-2m-4n\right){𝓇}^{5}{𝓉}^{5}+\left(24mn+4m-4n\right){𝓇}^{5}{𝓉}^{7}\\ \;\;\;\;\;\;\;\;\;\;\;\;\;\;\;\;\;\;\;\;\;\;\;\;\;\;\;\;\;+\left(6mn-21m+21n\right){𝓇}^{7}{𝓉}^{7} \end{array}$$

**Proof.**  *Let* $f\left(𝓇,𝓉\right)=NM\left(\Gamma ;𝓇,𝓉\right)=\left(12m+6n\right){𝓇}^{4}{𝓉}^{4}+\left(8m+4n\right){𝓇}^{4}{𝓉}^{5}+\left(12mn-2m-4n\right){𝓇}^{5}{𝓉}^{5}+\left(24mn+4m-4n\right){𝓇}^{5}{𝓉}^{7}+\left(6mn-21m+21n\right){𝓇}^{7}{𝓉}^{7}$

**Theorem** **5.** *If*Γ*is a β- graphyne system, then*NM-*polynomial of*Γ*is given as follows*

1. ${\mathrm{NM}}_{1}\left(\Gamma \right)=492\mathrm{mn}-98m+290n$
2. ${\mathrm{NM}}_{2}\left(\Gamma \right)=1434\mathrm{mn}-587m+965n$,
3. ${\mathrm{NM}}_{2}^{m}\left(\Gamma \right)=\left(\frac{529}{700}\right)m+\left(\frac{1021}{1400}\right)n+\left(\frac{1578}{1225}\right)\mathrm{mn}$,
4. ${\mathrm{NR}}_{\alpha }\left(\Gamma \right)=16^{\varphi }\left(12m+6n\right)+20^{\varphi }\left(8m+4n\right)+25^{\varphi }\left(12\mathrm{mn}-2m-4n\right)+35^{\varphi }\left(24\mathrm{mn}+4m-4n\right)+49^{\varphi }\left(6\mathrm{mn}-21m+21n\right)$,
5. ${\mathrm{NRR}}_{\alpha }\left(\Gamma \right)=\frac{1}{16^{\varphi }}\left(12m+6n\right)+\frac{1}{20^{\varphi }}\left(8m+4n\right)+\frac{1}{25^{\varphi }}\left(12\mathrm{mn}-2m-4n\right)+\frac{1}{35^{\varphi }}\left(24\mathrm{mn}+4m-4n\right)+\frac{1}{49^{\varphi }}\left(6\mathrm{mn}-21m+21n\right)$,
6. $\mathrm{NSDD}\left(\Gamma \right)=\left(\frac{20}{7}\right)m+\left(\frac{1601}{35}\right)n+\left(\frac{3036}{35}\right)\mathrm{mn}$,
7. $\mathrm{NH}\left(\Gamma \right)=\left(\frac{92}{45}\right)m+\left(\frac{353}{90}\right)n+\left(\frac{254}{35}\right)\mathrm{mn}$,
8. $\mathrm{NI}\left(\Gamma \right)=121\mathrm{mn}-\left(\frac{451}{18}\right)m+\left(\frac{1309}{18}\right)n$,
9. $\mathrm{NA}\left(\Gamma \right)=\left(\frac{2077879}{1152}\right)\mathrm{mn}-\left(\frac{715316939}{790272}\right)m+\left(\frac{530771995}{395136}\right)n$,
10. $\begin{array}{c} \mathrm{NABC}\left(\Gamma \right)=3\sqrt{2}\sqrt{3}\;m+\left(\frac{3}{2}\right)\sqrt{2}\sqrt{3}n+\left(\frac{4}{5}\right)\sqrt{7}\sqrt{5}\;m+\left(\frac{2}{5}\right)\sqrt{7}\sqrt{5}n+\left(\frac{24}{5}\right)\sqrt{2}\mathrm{mn}-\left(\frac{4}{5}\right)\sqrt{2}\;m-\left(\frac{8}{5}\right)\sqrt{2}n\\ +\left(\frac{24}{7}\right)\sqrt{2}\sqrt{7}\;m*n+\left(\frac{4}{7}\right)\sqrt{2}\sqrt{7}\;m-\left(\frac{4}{7}\right)\sqrt{2}\sqrt{7}n+\left(\frac{12}{7}\right)\sqrt{3}\mathrm{mn}-6\sqrt{3}\;m+6\sqrt{3}n \end{array}$,
11. $\mathrm{NGA}\left(\Gamma \right)=23n+\left(\frac{32}{9}\right)\sqrt{5}m+-11m+\left(\frac{16}{9}\right)\sqrt{5}n+18\mathrm{mn}+4\sqrt{7}\;\sqrt{5}\mathrm{mn}+\left(\frac{2}{3}\right)\sqrt{7}\;\sqrt{5}m-\left(\frac{2}{3}\right)\sqrt{7}\;\sqrt{5}n$,
12. ${\mathrm{NB}}_{1}\left(\Gamma \right)=1308\mathrm{mn}-298m+778n$,
13. ${\mathrm{NB}}_{2}\left(\Gamma \right)=4848\mathrm{mn}-2128m+3268n$,
14. ${\mathrm{NHB}}_{1}\left(\Gamma \right)=300672\mathrm{mn}-243256m+268900n$.

## 8. Computing Neighbourhood Degree Sum-Based Entropy Measures for β-graphyne

**Theorem** **6.** *If*Γ*is a β-Graphyne system, then*NM-*entropy measures of*Γ*are given as follows*

1. $\begin{array}{cc} NENT_{NM_{1}}\left(\Gamma \right)= & log(492mn-98m+290n)\\ \; & -\frac{1}{492mn-98m+290n}[\left(12m+6n\right)\left(8\right)log\left(8\right)+\left(8m+4n\right)\left(9\right)log9\\ \; & +(12mn-2m-4n)(10)log10+(24mn+4m-4n)(12)log12\\ \; & +(6mn-21m+21n)(14)log14] \end{array}$
  $=log\left(492mn-98m+290n\right)-\left(\frac{-344.8514m+743.4266n+1213.6476mn}{492mn-98m+290n}\right)$
2. $\begin{array}{cc} NENT_{NM_{2}^{m}}\left(\Gamma \right)= & log(1434mn-587m+965n)\\ \; & -\frac{1}{1434mn-587m+965n}[\left(12m+6n\right)\left(16\right)log16+\left(8m+4n\right)\left(20\right)log20\\ \; & +(12mn-2m-4n)(25)log25+(24mn+4m-4n)(35)log35\\ \; & +(6mn-21m+21n)(49)log49] \end{array}$
  $=log\left(1434mn-587m+965n\right)-\left(\frac{-2656.2090m+3690.8658n+5096.2812mn}{1434mn-587m+965n}\right)$
3. $\begin{array}{cc} NENT_{NM_{2}^{m}}\left(\Gamma \right)= & log\left(\left(\frac{529}{700}\right)m+\left(\frac{1021}{1400}\right)n+\left(\frac{1578}{1225}\right)mn\right)\\ \; & -\frac{1}{\left(\frac{529}{700}\right)m+\left(\frac{1021}{1400}\right)n+\left(\frac{1578}{1225}\right)mn}\left[\left(12m+6n\right)\left(\frac{1}{16}\right)log\frac{1}{16}\right.\\ \; & +\left(8m+4n\right)\left(\frac{1}{20}\right)log\frac{1}{20}+\left(12mn-2m-4n\right)\left(\frac{1}{25}\right)log\frac{1}{25}\\ \; & \left.+\left(24mn+4m-4n\right)\left(\frac{1}{35}\right)log\frac{1}{35}+\left(6mn-21m+21n\right)\left(\frac{1}{49}\right)log\frac{1}{49}\right] \end{array}$
  $=log\left(\left(\frac{529}{700}\right)m+\left(\frac{1021}{1400}\right)n+\left(\frac{1578}{1225}\right)mn\right)-\left(\frac{-1.758631714m-2.385451286n-4.459490939mn}{\left(\frac{529}{700}\right)m+\left(\frac{1021}{1400}\right)n+\left(\frac{1578}{1225}\right)mn}\right)$
4. $\begin{array}{cc} NENT_{NHM}\left(\Gamma \right)= & log(5832mn-2328m+2220n+84)\\ \; & -\frac{1}{5832mn-2328m+2220n+84}[\left(12m+6n\right)\left(64\right)log64+\left(8m+4n\right)\left(81\right)log81\\ \; & +(12mn-2m-4n)(100)log100+(24mn+4m-4n)(144)log144\\ \; & +(6mn-21m+21n)(196)log196] \end{array}$
  $=log\left(5832mn-2328m+2220n+84\right)-\left(\frac{-13741.9000m+20041.1896n+28909.0320mn}{5832mn-2328m+2220n+84}\right)$
5. $\begin{array}{cc} NENT_{NA}\left(\Gamma \right)= & log\left(\left(\frac{2077879}{1152}\right)mn+\left(\frac{530771995}{395136}\right)n-\left(\frac{715316939}{790272}\right)m\right)\\ \; & -\frac{1}{\left(\frac{2077879}{1152}\right)mn+\left(\frac{530771995}{395136}\right)n-\left(\frac{715316939}{790272}\right)m}\left[\left(12m+6n\right){\left(\frac{16}{6}\right)}^{3}log\frac{16^{3}}{6}\right.\\ \; & +\left(8m+4n\right){\left(\frac{20}{7}\right)}^{3}log{\frac{20}{7}}^{3}+\left(12mn-2m-4n\right){\left(\frac{25}{8}\right)}^{3}log\frac{25^{3}}{8}\\ \; & \left.+\left(24mn+4m-4n\right){\left(\frac{35}{10}\right)}^{3}log\frac{35^{3}}{10}+\left(6mn-21m+21n\right){\left(\frac{49}{12}\right)}^{3}log\frac{49^{3}}{12}\right] \end{array}$
  $=log\left(\left(\frac{2077879}{1152}\right)mn+\left(\frac{530771995}{395136}\right)n-\left(\frac{715316939}{790272}\right)m\right)-\left(\frac{-3304.057315m+3679.118326n+3702.622388mn}{\left(\frac{2077879}{1152}\right)mn+\left(\frac{530771995}{395136}\right)n-\left(\frac{715316939}{790272}\right)m}\right)$
6. $\begin{array}{cc} NENT_{NABC}\left(\Gamma \right)= & log(5832mn-2328m+2220n+84)\\ \; & -\frac{1}{5832mn-2328m+2220n+84}\left[\left(12m+6n\right)\left(\sqrt{\frac{6}{16}}\right)log\sqrt{\frac{6}{16}}\right.\\ \; & +\left(8m+4n\right)\left(\sqrt{\frac{7}{20}}\right)log\sqrt{\frac{7}{20}}+\left(12mn-2m-4n\right)\left(\sqrt{\frac{8}{25}}\right)log\sqrt{\frac{8}{25}}\\ \; & \left.+\left(24mn+4m-4n\right)\left(\sqrt{\frac{10}{35}}\right)log\sqrt{\frac{10}{35}}+\left(6mn-21m+21n\right)\left(\sqrt{\frac{12}{49}}\right)log\sqrt{\frac{12}{49}}\right] \end{array}$
7. $\begin{array}{c} NENT_{NGA}\left(\Gamma \right)\\ =log\left(23n+\left(\frac{32}{9}\right)\sqrt{5}m+\left(\frac{16}{9}\right)\sqrt{5}n+18mn+4\sqrt{5}\sqrt{7}mn+\left(\frac{2}{3}\right)\sqrt{7}\sqrt{5}m-\left(\frac{2}{3}\right)\sqrt{7}\sqrt{5}n-11m\right)\\ -\frac{1}{23n+\left(\frac{32}{9}\right)\sqrt{5}m+\left(\frac{16}{9}\right)\sqrt{5}n+18mn+4\sqrt{5}\sqrt{7}mn+\left(\frac{2}{3}\right)\sqrt{7}\sqrt{5}m-\left(\frac{2}{3}\right)\sqrt{7}\sqrt{5}n-11m}[(12m\\ +6n)\left(1\right)log1+\left(8m+4n\right)\left(\frac{2\sqrt{20}}{9}\right)log\frac{2\sqrt{20}}{9}+\left(12mn-2m-4n\right)\left(1\right)log1\\ \left.+\left(24mn+4m-4n\right)\left(\frac{2\sqrt{35}}{12}\right)log\frac{2\sqrt{35}}{12}+\left(6mn-21m+21n\right)\left(1\right)log1\right] \end{array}$
8. $\begin{array}{c} NENT_{NAG_{1}}\left(\Gamma \right)\\ =log\left(23n+\left(\frac{18}{5}\right)\sqrt{5}m+\left(\frac{9}{5}\right)\sqrt{5}n+18mn+\left(\frac{144}{35}\right)\sqrt{5}\sqrt{7}mn+\left(\frac{24}{35}\right)\sqrt{5}\sqrt{7}m-\left(\frac{24}{35}\right)\sqrt{7}\sqrt{5}n-11m\right)\\ -\frac{1}{23n+\left(\frac{18}{5}\right)\sqrt{5}m+\left(\frac{9}{5}\right)\sqrt{5}n+18mn+\left(\frac{144}{35}\right)\sqrt{5}\sqrt{7}mn+\left(\frac{24}{35}\right)\sqrt{5}\sqrt{7}m-\left(\frac{24}{35}\right)\sqrt{7}\sqrt{5}n-11m}[(12m\\ +6n)\left(1\right)log1+\left(8m+4n\right)\left(\frac{9}{2\sqrt{20}}\right)log\frac{9}{2\sqrt{20}}+\left(12mn-2m-4n\right)\left(1\right)log1\\ \left.+\left(24mn+4m-4n\right)\left(\frac{12}{2\sqrt{35}}\right)log\frac{12}{2\sqrt{35}}+\left(6mn-21m+21n\right)\left(1\right)log1\right] \end{array}$
9. $\begin{array}{cc} NENT_{NF_{1}}\left(\Gamma \right)= & log(2964mn-1150m+1918n)\\ \; & -\frac{1}{2964mn-1150m+1918n}[\left(12m+6n\right)\left(32\right)log\left(32\right)+\left(8m+4n\right)\left(41\right)log41\\ \; & +(12mn-2m-4n)(50)log50+(24mn+4m-4n)(74)log74\\ \; & +(6mn-21m+21n)(98)log98] \end{array}$
  $=log\left(2964mn-1150m+1918n\right)-\left(\frac{-3256.8298m+5906.6218n+11902.3020mn}{2964mn-1150m+1918n}\right)$
10. $\begin{array}{cc} NENT_{NF_{2}}\left(\Gamma \right)= & log(49146mn-32939m+38597n)\\ \; & -\frac{1}{49146mn-32939m+38597n}[\left(12m+6n\right)\left(256\right)log\left(256\right)\\ \; & +(8m+4n)(400)log400+(12mn-2m-4n)(625)log625\\ \; & +(24mn+4m-4n)(1225)log1225+(6mn-21m+21n)(2401)log2401] \end{array}$
  $=\log\left(49146mn-32939m+38597n\right)-\left(\frac{-3.294546212\times m+3.596246228\times 10^{5}n+3.694641816\times 10^{5}mn}{49146mn-32939m+38597n}\right)$
11. $\begin{array}{c} NENT_{N\chi }\left(\Gamma \right)=log\left(\chi \left(\Gamma \right)\right)\\ -\frac{1}{\chi (\Gamma )}\left[\left(12m+6n\right)\left(\frac{1}{\sqrt{8}}\right)log\frac{1}{\sqrt{8}}+\left(8m+4n\right)\left(\frac{1}{\sqrt{9}}\right)log\frac{1}{\sqrt{9}}\right.\\ +\left(12mn-2m-4n\right)\left(\frac{1}{\sqrt{10}}\right)log\frac{1}{\sqrt{10}}+\left(24mn+4m-4n\right)\left(\frac{1}{\sqrt{12}}\right)log\frac{1}{\sqrt{12}}\\ \left.+\left(6mn-21m+21n\right)\left(\frac{1}{\sqrt{14}}\right)log\frac{1}{\sqrt{14}}\right] \end{array}$
12. $\begin{array}{cc} NENT_{NReZ\Gamma_{1}}\left(\Gamma \right)= & log\left(\left(\frac{146}{35}\right)m+\left(\frac{274}{35}\right)n+\left(\frac{516}{35}\right)mn\right)\\ \; & -\frac{1}{\left(\frac{146}{35}\right)m+\left(\frac{274}{35}\right)n+\left(\frac{516}{35}\right)mn}\left[\left(12m+6n\right)\left(\frac{8}{16}\right)log\frac{8}{16}+\left(8m+4n\right)\left(\frac{9}{20}\right)log\frac{9}{20}\right.\\ \; & +\left(12mn-2m-4n\right)\left(\frac{10}{25}\right)log\frac{10}{25}+\left(24mn+4m-4n\right)\left(\frac{12}{35}\right)log\frac{12}{35}\\ \; & \left.+\left(6mn-21m+21n\right)\left(\frac{14}{49}\right)log\frac{14}{49}\right] \end{array}$
  $=log\left(\left(\frac{146}{35}\right)m+\left(\frac{274}{35}\right)n+\left(\frac{516}{35}\right)mn\right)-\left(\frac{-.251681144m-8.099526856n-15.35371200mn}{\left(\frac{146}{35}\right)m+\left(\frac{274}{35}\right)n+\left(\frac{516}{35}\right)mn}\right)$
13. $\begin{array}{cc} {\mathrm{NENT}}_{{N\mathrm{NReZG}}_{2}}\left(\Gamma \right)= & log\left(\left(\frac{1309}{18}\right)n+121mn-\left(\frac{451}{18}\right)m\right)\\ \; & -\frac{1}{\left(\frac{1309}{18}\right)n+121mn-\left(\frac{451}{18}\right)m}\left[\left(12m+6n\right)\left(\frac{16}{8}\right)log\frac{16}{8}+\left(8m+4n\right)\left(\frac{20}{9}\right)log\frac{20}{9}\right.\\ \; & +\left(12mn-2m-4n\right)\left(\frac{25}{10}\right)log\frac{25}{10}+\left(24mn+4m-4n\right)\left(\frac{35}{12}\right)log\frac{35}{12}\\ \; & \left.+\left(6mn-21m+21n\right)\left(\frac{49}{14}\right)log\frac{49}{14}\right] \end{array}$
  $=log\left(\left(\frac{1309}{18}\right)n+121mn-\left(\frac{451}{18}\right)m\right)-\left(\frac{-53.34291666m+85.84556667n+128.7255000mn}{\left(\frac{1309}{18}\right)n+121mn-\left(\frac{451}{18}\right)m}\right)$
14. $\begin{array}{cc} NENT_{NReZG_{3}}\left(\Gamma \right)= & log(17196mn-10250m+13214n)\\ \; & -\frac{1}{17196mn-10250m+13214n}[\left(12m+6n\right)\left(128\right)log128\\ \; & +(8m+4n)(180)log180+(12mn-2m-4n)(250)log250\\ \; & +(24mn+4m-4n)(420)log420+(6mn-21m+21n)(686)log686] \end{array}$
  $=log\left(17196mn-10250m+13214n\right)-\left(\frac{-71766.5994m+85880.2374n+1.043319084\times 10^{5}mn}{17196mn-10250m+13214n}\right)$

where $\begin{array}{c} NABC\left(\Gamma \right)=3\sqrt{2}\sqrt{3}m+\left(\frac{3}{2}\right)\sqrt{2}\sqrt{3}n+\left(\frac{4}{5}\right)\sqrt{7}\sqrt{5}m+\left(\frac{2}{5}\right)\sqrt{7}\sqrt{5}n+\left(\frac{24}{5}\right)\sqrt{2}mn-\left(\frac{4}{5}\right)\sqrt{2}m\\ -\left(\frac{8}{5}\right)\sqrt{2}n+\left(\frac{24}{7}\right)\sqrt{2}\sqrt{7}mn+\left(\frac{4}{7}\right)\sqrt{2}\sqrt{7}m-\left(\frac{4}{7}\right)\sqrt{2}\sqrt{7}n+\left(\frac{12}{7}\right)\sqrt{3}mn-6\sqrt{3}m+6\sqrt{3}n \end{array}$ and $\begin{array}{c} \mathbb{N}\chi \left(\Gamma \right)=3\sqrt{2}m+\left(\frac{3}{2}\right)\sqrt{2}n+\left(\frac{8}{3}\right)m+\left(\frac{4}{3}\right)n+\left(\frac{6}{5}\right)\sqrt{5}\sqrt{2}mn-\left(\frac{1}{5}\right)\sqrt{5}\sqrt{2}m\\ -\left(\frac{2}{5}\right)\sqrt{5}\sqrt{2}n+4\sqrt{3}mn+\left(\frac{2}{3}\right)\sqrt{3}m-\left(\frac{2}{3}\right)\sqrt{3}n+\left(\frac{3}{7}\right)\sqrt{2}\sqrt{7}mn-\left(\frac{3}{2}\right)\sqrt{2}\sqrt{7}m+\left(\frac{3}{2}\right)\sqrt{2}\sqrt{7}n \end{array}$.

**Figure 8:**
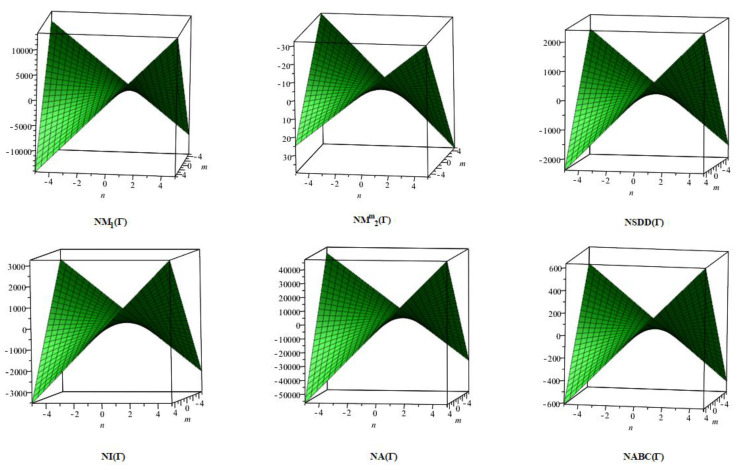

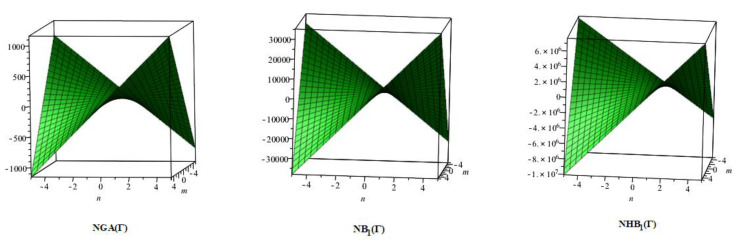
3D plots for theorem 5.

**Figure 9:**
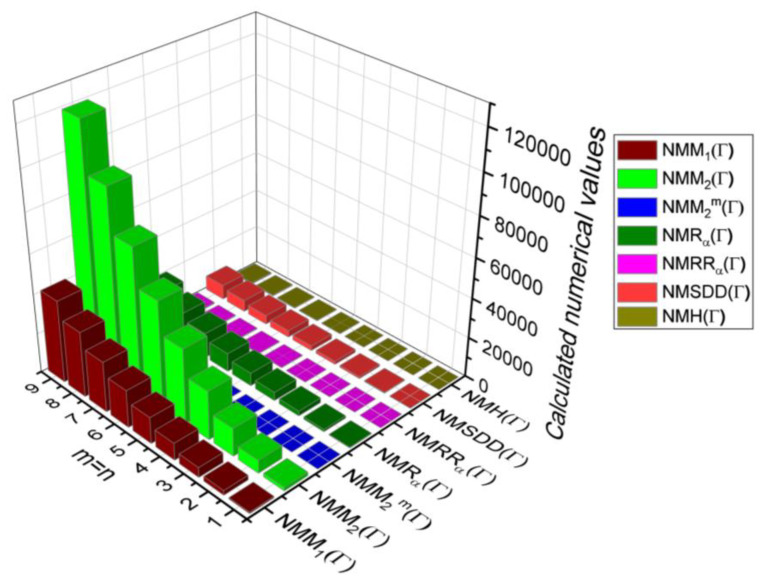
3D plots for Table 8.

**Figure 10:**
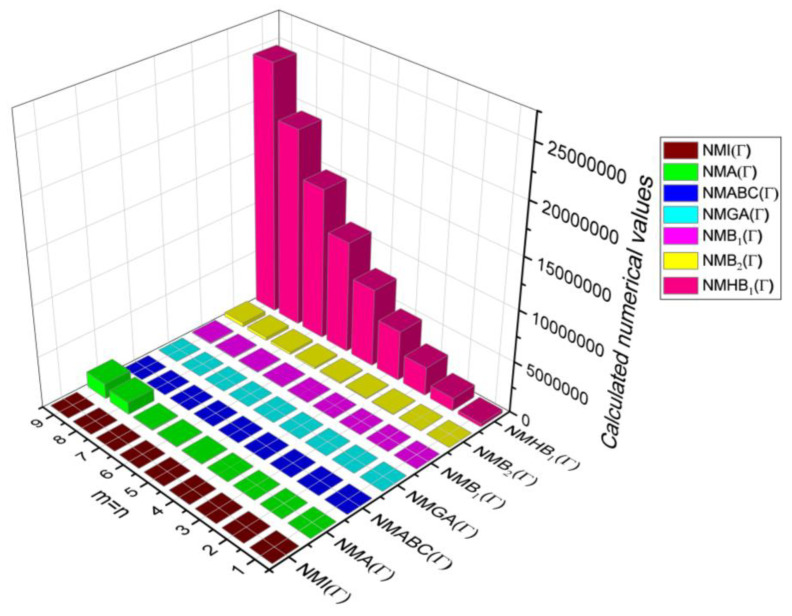
3D plots for Table 9.

**Figure 11:**
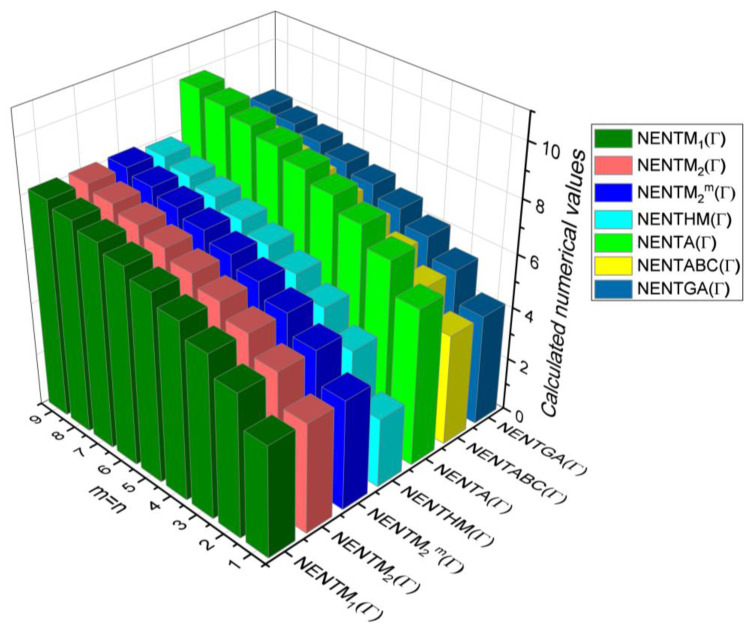
3D plots for Table 10.

**Figure 12:**
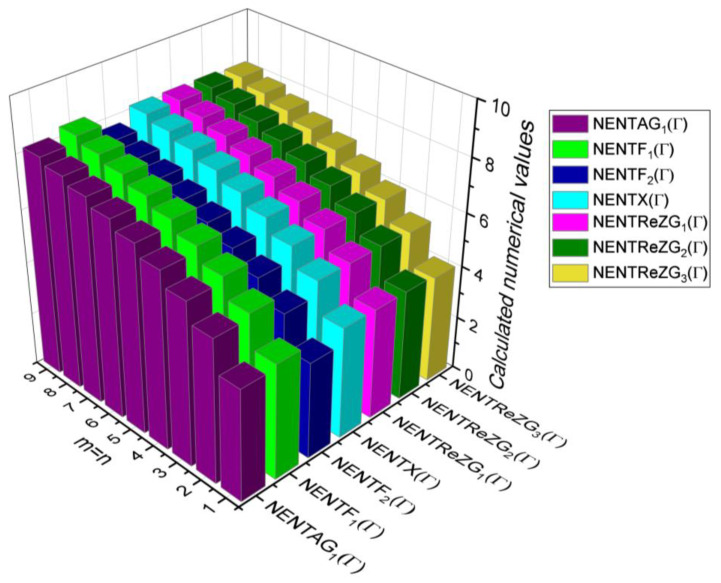
3D plots for Table 11.

The closed-form expressions of the neighbourhood degree sum-based indices computed in Theorem 5 and Theorem 6 are represented as 3D plots in this section. These graphical representations provide a visual interpretation of the mathematical expressions to study the indices with respect to the molecular structure. In addition, the results of Theorem 2 have also been represented as comparison plots to understand the behaviour of the indices with respect to each other. These plots are presented in Figure 8, Figure 9, Figure 10, Figure 11 and Figure 12.

## 9. Neighbourhood Degree Sum-Based M-polynomial and Entropy Measures for β-Graphdiyne

**Figure 13:**
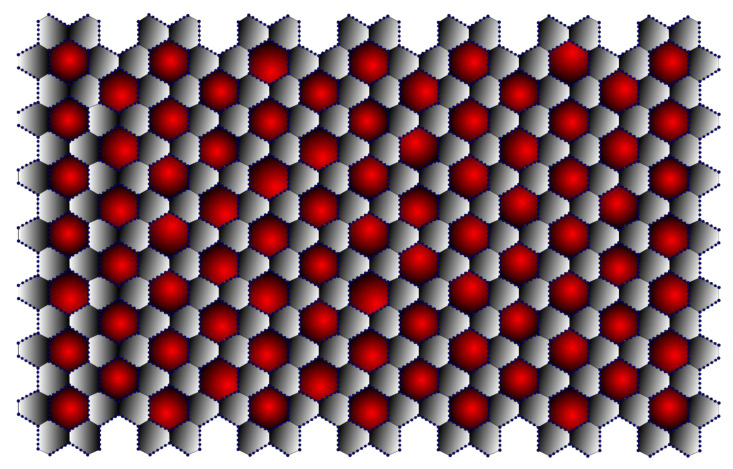
β-Graphdiyne (7,7).

Graphdiyne, a recently developed two-dimensional carbon allotrope, has been gaining prominence. This carbon allotrope has gained increasing interest due to its intriguing electronic properties, including high mobility and conductivity, good field emission properties, and a tunable natural band gap, in addition to its distinctive porous structure. It is predicted to have good application prospects in a variety of fields, including gas separation, catalysis, water remediation, humidity sensor, and energy-related fields [33]. It consists of V(Γ)=60mn+2m+34n and E(Γ)=66nm+35m+n. β-Graphdiyne is represented in Figure 13. The edge partitions of β-Graphdiyne are presented in Table 12.

Recently, Julietraja and Venugopal [34] studied degree-based indices using M-Polynomials for a coronoid system. Chu et al. [35] studied degree-and irregularity-based indices for benzenoids. Julietraja et al. [36] investigated degree-based indices for certain classes of benzenoid systems. Julietraja et al. [37] investigated degree-based indices for a donut-benzenoid system. Julietraja et al. [38] investigated irregularity-based indices for PAHs. Liu et al. [39] studied the network coherence analysis on a family of nested weighted N-Polygon networks. Liu et al. [40] studied the analyses of some structural properties on a class of hierarchical scale-free networks. Liu et al. [41] studied minimizing the Kirchhoff index among graphs with a given vertex bipartiteness. Liu et al. [42] studied the Zagreb indices and multiplicative Zagreb indices of Eulerian Graphs. Liu et al. [43] studied the valency-based topological descriptors and structural properties of the generalized Sierpinski networks. Liu et al. [44] studied the Kirchhoff index and spanning trees of the Möbius/cylinder octagonal chain.

**Theorem** **7.** *If*Γ*is a β- graphdiyne system, then*NM-*polynomial of*Γ*is given as follows*

$$\begin{array}{l} NM\left(\Gamma ;𝓇,𝓉\right)=\left(12mn+26m+10n\right){𝓇}^{4}{𝓉}^{4}+\left(24mn+4m-4n\right){𝓇}^{4}{𝓉}^{5}\\ \;\;\;\;\;\;\;\;\;\;\;\;\;\;\;\;\;\;\;\;\;\;\;\;\;\;\;+\left(24mn+4m-4n\right){𝓇}^{5}{𝓉}^{7}+\left(6mn+m-n\right){𝓇}^{7}{𝓉}^{7} \end{array}$$

**Proof.**  *Let* $f\left(𝓇,𝓉\right)=NM\left(\Gamma ;𝓇,𝓉\right)=\left(12mn+26m+10n\right){𝓇}^{4}{𝓉}^{4}+\left(24mn+4m-4n\right){𝓇}^{4}{𝓉}^{5}+\left(24mn+4m-4n\right){𝓇}^{5}{𝓉}^{7}+\left(6mn+m-n\right){𝓇}^{7}{𝓉}^{7}$

**Theorem** **8.** *If*Γ*is a β- graphdiyne system, then*NM-*entropy measures of*Γ*are given as follows*

1. $NM_{1}\left(\Gamma \right)=684mn+306m-18n$
2. $NM_{2}\left(\Gamma \right)=1806mn+685m-109n$
3. $NM_{2}^{m}\left(\Gamma \right)=\left(\frac{2703}{980}\right)mn+\left(\frac{3841}{1960}\right)m+\left(\frac{569}{1960}\right)n$
4. $NR_{\alpha }\left(\Gamma \right)=16^{\varphi }\left(12mn+26m+10n\right)+20^{\varphi }\left(24mn+4m-4n\right)+35^{\varphi }\left(24mn+4m-4n\right)+49^{\varphi }\;\left(6mn+m-n\right)$
5. $NRR_{\alpha }\left(\Gamma \right)=1/16^{\varphi }\;\left(12mn+26m+10n\right)+1/20^{\varphi }\;\left(24mn+4m-4n\right)+1/35^{\varphi }\left(24mn+4m-4n\right)+1/49^{\varphi }\;\left(6mn+m-n\right)$
6. $NSDD\left(\Gamma \right)=\left(\frac{4758}{35}\right)mn+\left(\frac{2473}{35}\right)m+\left(\frac{47}{35}\right)n$
7. $NH\left(\Gamma \right)=\left(\frac{277}{21}\right)mn+\left(\frac{1033}{126}\right)m+\left(\frac{101}{126}\right)n$
8. $NI\left(\Gamma \right)=\left(\frac{505}{3}\right)mn+\left(\frac{1369}{18}\right)m-\left(\frac{73}{18}\right)n$
9. $NA\left(\Gamma \right)=\left(\frac{219777191}{98784}\right)mn+\left(\frac{489523367}{592704}\right)m-\left(\frac{84904103}{592704}\right)n$
10. $\begin{array}{c} NABC\left(\Gamma \right)=3\sqrt{3}\sqrt{2}mn+\left(\frac{13}{2}\right)\sqrt{3}\sqrt{2}m+\left(\frac{5}{2}\right)\sqrt{3}\sqrt{2}n+\left(\frac{12}{5}\right)\sqrt{7}\sqrt{5}mn+\left(\frac{2}{5}\right)\sqrt{7}\sqrt{5}m-\left(\frac{2}{5}\right)\sqrt{7}\sqrt{5}n\\ +\left(\frac{24}{7}\right)\sqrt{2}\sqrt{7}mn+\left(\frac{4}{7}\right)\sqrt{2}\sqrt{7}m-\left(\frac{4}{7}\right)\sqrt{2}\sqrt{7}n+\left(\frac{12}{7}\right)\sqrt{3}mn+\left(\frac{2}{7}\right)\sqrt{3}m-\left(\frac{2}{7}\right)\sqrt{3}n \end{array}$
11. $NGA\left(\Gamma \right)=18mn+27m+9n+\left(\frac{32}{3}\right)\;\sqrt{5}mn+\left(\frac{16}{9}\right)\;\sqrt{5}m-\left(\frac{16}{9}\right)\;\sqrt{5}n+4\sqrt{7}\;\sqrt{5}mn+\left(\frac{2}{3}\right)\sqrt{7}\;\sqrt{5}m-\left(\frac{2}{3}\right)\sqrt{7}\;\sqrt{5}n$
12. $NB_{1}\left(\Gamma \right)=1788mn+778m-58n$
13. $NB_{2}\left(\Gamma \right)=5016mn+1988m-260n$
14. $NHB_{1}\left(\Gamma \right)=324312mn+81700m-40228n$

## 10. Neighbourhood Degree Sum-Based Entropy Measures for β-Graphdiyne

**Theorem** **9.** *If*Γ*is a β- graphdiyne system, then*NM-*entropy measures of*Γ*are given as follows*

1. $\begin{array}{cc} NENT_{NM_{1}}\left(\Gamma \right)= & log(684mn+306m-18n)\\ \; & -\frac{1}{684mn+306m-18n}[\left(12mn+26m+10n\right)\left(8\right)log8\\ \; & +(24mn+4m-4n)(9)log9+(24mn+4m-4n)(12)log12\\ \; & +(6mn+m-n)(14)log14] \end{array}$
  $=\log\left(684mn+306m-18n\right)-\left(\frac{1611.5532mn+667.8370m-68.9698n}{684mn+306m-18n}\right)$
2. $\begin{array}{cc} NENT_{NM_{2}}\left(\Gamma \right)= & log(1806mn+685m-109n)\\ \; & -\frac{1}{1806mn+685m-109n}[\left(12mn+26m+10n\right)\left(16\right)log16\\ \; & +(24mn+4m-4n)(20)log20+(24mn+4m-4n)(35)log35\\ \; & +(6mn+m-n)(49)log49] \end{array}$
  $=log\left(1806mn+685m-109n\right)-\left(\frac{6100.9164mn+2081.4978m-484.4802n}{1806mn+685m-109n}\right)$
3. $\begin{array}{cc} NENT_{NM_{2}^{m}}\left(\Gamma \right)= & log\left(\left(\frac{2703}{980}\right)mn+\left(\frac{3841}{1960}\right)m+\left(\frac{569}{1960}\right)n\right)\\ \; & -\frac{1}{\left(\frac{2703}{980}\right)mn+\left(\frac{3841}{1960}\right)m+\left(\frac{569}{1960}\right)n}\left[\left(12mn+26m+10n\right)\left(\frac{1}{16}\right)log\frac{1}{16}\right.\\ \; & +\left(24mn+4m-4n\right)\left(\frac{1}{20}\right)log\frac{1}{20}+\left(24mn+4m-4n\right)\left(\frac{1}{35}\right)log\frac{1}{35}\\ \; & \left.+\left(6mn+m-n\right)\left(\frac{1}{49}\right)log\frac{1}{49}\right] \end{array}$
  $=log\left(\left(\frac{2703}{980}\right)mn+\left(\frac{3841}{1960}\right)m+\left(\frac{569}{1960}\right)n\right)-\left(\frac{-8.588756939mn-5.590359490m-.6479905100n}{\left(\frac{2703}{980}\right)mn+\left(\frac{3841}{1960}\right)m+\left(\frac{569}{1960}\right)n}\right)$
4. $\begin{array}{cc} NENT_{NHM}\left(\Gamma \right)= & log(7344mn+2760m-456n)\\ \; & -\frac{1}{7344mn+2760m-456n}[\left(12mn+26m+10n\right)\left(64\right)log64\\ \; & +(24mn+4m-4n)(81)log81+(24mn+4m-4n)(144)log144\\ \; & +(6mn+m-n)(196)log196] \end{array}$
  $=log\left(7344mn+2760m-456n\right)-\left(\frac{-35119.5408mn+12241.3272m-2659.2216n}{7344mn+2760m-456n}\right)$
5. $\begin{array}{cc} NENT_{NA}\left(\Gamma \right)= & log\left(\left(\frac{219777191}{98784}\right)mn+\left(\frac{489523367}{592704}\right)m-\left(\frac{84904103}{592704}\right)n\right)\\ \; & -\frac{1}{\left(\frac{219777191}{98784}\right)mn+\left(\frac{489523367}{592704}\right)m-\left(\frac{84904103}{592704}\right)n}[(12mn+26m\\ \; & +10n){\left(\frac{16}{6}\right)}^{3}log\frac{16^{3}}{6}+\left(24mn+4m-4n\right){\left(\frac{20}{7}\right)}^{3}log\frac{20^{3}}{7}\\ \; & \left.+\left(24mn+4m-4n\right){\left(\frac{35}{10}\right)}^{3}log\frac{35^{2}}{10}+\left(6mn+m-n\right){\left(\frac{49}{12}\right)}^{3}log\frac{49^{3}}{12}\right] \end{array}$
  $=log\left(\left(\frac{219777191}{98784}\right)mn+\left(\frac{489523367}{592704}\right)m-\left(\frac{84904103}{592704}\right)n\right)-\left(\frac{4023.297013mn+1099.996898m-455.8258044n}{\left(\frac{219777191}{98784}\right)mn+\left(\frac{489523367}{592704}\right)m-\left(\frac{84904103}{592704}\right)n}\right)$
6. $\begin{array}{cc} NENT_{NABC}\left(\Gamma \right)= & log(NABC(\Gamma ))\\ \; & -\frac{1}{NABC(\Gamma )}\left[\left(12mn+26m+10n\right)\left(\sqrt{\frac{6}{16}}\right)log\sqrt{\frac{6}{16}}\right.\\ \; & +\left(24mn+4m-4n\right)\left(\sqrt{\frac{7}{20}}\right)log\sqrt{\frac{7}{20}}+\left(24mn+4m-4n\right)\left(\sqrt{\frac{10}{35}}\right)log\sqrt{\frac{10}{35}}\\ \; & \left.+\left(6mn+m-n\right)\left(\sqrt{\frac{12}{49}}\right)log\sqrt{\frac{12}{49}}\right] \end{array}$
7. $\begin{array}{cc} NENT_{NGA}\left(\Gamma \right)= & log(NGA(\Gamma ))\\ \; & -\frac{1}{NGA(\Gamma )}\left[\left(12mn+26m+10n\right)\left(1\right)log1+\left(24mn+4m-4n\right)\left(\frac{2\sqrt{20}}{9}\right)log\frac{2\sqrt{20}}{9}\right.\\ \; & \left.+\left(24mn+4m-4n\right)\left(\frac{2\sqrt{35}}{12}\right)log\frac{2\sqrt{35}}{12}+\left(6mn+m-n\right)\left(1\right)log1\right] \end{array}$
8. $\begin{array}{cc} NENT_{NAG_{1}}\left(\Gamma \right)= & log\left(NAG_{1}\left(\Gamma \right)\right)\\ \; & -\frac{1}{NAG_{1}\left(\Gamma \right)}\left[\left(12mn+26m+10n\right)\left(1\right)log1+\left(24mn+4m-4n\right)\left(\frac{9}{2\sqrt{20}}\right)log\frac{9}{2\sqrt{20}}\right.\\ \; & \left.+\left(24mn+4m-4n\right)\left(\frac{12}{2\sqrt{35}}\right)log\frac{12}{2\sqrt{35}}+\left(6mn+m-n\right)\left(1\right)log1\right] \end{array}$
9. $\begin{array}{cc} NENT_{NF_{1}}\left(\Gamma \right)= & log(3732mn+1390m-238n)\\ \; & -\frac{1}{3732mn+1390m-238n}[\left(12mn+26m+10n\right)\left(32\right)log32\\ \; & +(24mn+4m-4n)(41)log41+(24mn+4m-4n)(74)log74\\ \; & +(6mn+m-n)(98)log98] \end{array}$
  $=log\left(3732mn+1390m-238n\right)-\left(\frac{15325.1112mn+5215.9196m-1223.3180n}{3732mn+1390m-238n}\right)$
10. $\begin{array}{cc} NENT_{NF_{2}}\left(\Gamma \right)= & log(54318mn+15197m-5981n)\\ \; & -\frac{1}{54318mn+15197m-5981n}[\left(12mn+26m+10n\right)\left(256\right)log256\\ \; & +(24mn+4m-4n)(400)log400+(24mn+4m-4n)(1225)log1225\\ \; & +(6mn+m-n)(2401)log2401] \end{array}$
  $=log\left(54318mn+15197m-5981n\right)-\left(\frac{3.957344760\times 10^{5}mn+1.000254548\times 10^{5}m-48920.8916n}{54318mn+15197m-5981n}\right)$
11. $\begin{array}{cc} NENT_{N\chi }\left(\Gamma \right)= & log(\chi (\Gamma ))\\ \; & -\frac{1}{\chi (\Gamma )}\left[\left(12mn+26m+10n\right)\left(\frac{1}{\sqrt{8}}\right)log\frac{1}{\sqrt{8}}+\left(24mn+4m-4n\right)\left(\frac{1}{3}\right)log\frac{1}{3}\right.\\ \; & \left.+\left(24mn+4m-4n\right)\left(\frac{1}{\sqrt{12}}\right)log\frac{1}{\sqrt{12}}+\left(6mn+m-n\right)\left(\frac{1}{\sqrt{14}}\right)log\frac{1}{\sqrt{14}}\right] \end{array}$
12. $\begin{array}{cc} NENT_{NReZ\Gamma_{1}}\left(\Gamma \right)= & log\left(\left(\frac{936}{35}\right)mn+\left(\frac{576}{35}\right)m+\left(\frac{54}{35}\right)n\right)\\ \; & -\frac{1}{\left(\frac{936}{35}\right)mn+\left(\frac{576}{35}\right)m+\left(\frac{54}{35}\right)n}\left[\left(12mn+26m+10n\right)\left(\frac{1}{2}\right)log\frac{1}{2}\right.\\ \; & +\left(24mn+4m-4n\right)\left(\frac{9}{20}\right)log\frac{9}{20}+\left(24mn+4m-4n\right)\left(\frac{12}{35}\right)log\frac{12}{35}\\ \; & \left.+\left(6mn+m-n\right)\left(\frac{2}{7}\right)log\frac{2}{7}\right] \end{array}$
  $=log\left(\left(\frac{936}{35}\right)mn+\left(\frac{576}{35}\right)m+\left(\frac{54}{35}\right)n\right)-\left(\frac{-23.73832800mn-12.27418800m-.202512000n}{\left(\frac{936}{35}\right)mn+\left(\frac{576}{35}\right)m+\left(\frac{54}{35}\right)n}\right)$
13. $\begin{array}{cc} NENT_{NReZG_{2}}\left(\Gamma \right)= & log\left(\left(\frac{505}{3}\right)mn+\left(\frac{1369}{18}\right)m-\left(\frac{73}{18}\right)n\right)\\ \; & -\frac{1}{\left(\frac{505}{3}\right)mn+\left(\frac{1369}{18}\right)m-\left(\frac{73}{18}\right)n}[\left(12mn+26m+10n\right)\left(2\right)log2\\ \; & +\left(24mn+4m-4n\right)\left(\frac{20}{9}\right)log\frac{20}{9}+\left(24mn+4m-4n\right)\left(\frac{35}{12}\right)log\frac{35}{12}\\ \; & \left.+\left(6mn+m-n\right)\left(\frac{7}{2}\right)log\left(\frac{7}{2}\right)\right] \end{array}$
  $=log\left(\left(\frac{505}{3}\right)mn+\left(\frac{1369}{18}\right)m-\left(\frac{73}{18}\right)n\right)-\left(\frac{160.4596000mn+60.01446667m-10.10766667n}{\left(\frac{505}{3}\right)mn+\left(\frac{1369}{18}\right)m-\left(\frac{73}{18}\right)n}\right)$
14. $\begin{array}{cc} NENT_{NReZG_{3}}\left(\Gamma \right) & =log(20052mn+6414m-1806n)\\ \; & -\frac{1}{20052mn+6414m-1806n}[\left(12mn+26m+10n\right)\left(128\right)log128\\ \; & +(24mn+4m-4n)(180)log180+(24mn+4m-4n)(420)log420\\ \; & +(6mn+m-n)(686)log686] \end{array}$
  $=log\left(20052mn+6414m-1806n\right)-\left(\frac{1.176538404\times 10^{5}mn+34514.3174m-12156.3014n}{20052mn+6414m-1806n}\right)$

where $\begin{array}{c} NABC\left(\Gamma \right)=3\sqrt{3}\sqrt{2}mn+\left(\frac{13}{2}\right)\sqrt{3}\sqrt{2}m+\left(\frac{5}{2}\right)\sqrt{3}\sqrt{2}n+\frac{49}{14}\left(\frac{12}{5}\right)\sqrt{7}\sqrt{5}mn+\left(\frac{2}{5}\right)\sqrt{7}\sqrt{5}m\\ -\left(\frac{2}{5}\right)\sqrt{7}\sqrt{5}n+\left(\frac{24}{7}\right)\sqrt{2}\sqrt{7}mn+\left(\frac{4}{7}\right)\sqrt{2}\sqrt{7}m-\left(\frac{4}{7}\right)\sqrt{2}\sqrt{7}n+\left(\frac{12}{7}\right)\sqrt{3}mn+\left(\frac{2}{7}\right)\sqrt{3}m-\left(\frac{2}{7}\right)\sqrt{3}n \end{array}$, $NGA\left(\Gamma \right)=18mn+27m+9n+\left(\frac{32}{3}\right)\sqrt{5}mn+\left(\frac{16}{9}\right)\sqrt{5}m-\left(\frac{16}{9}\right)\sqrt{5}n+4\sqrt{7}\sqrt{5}mn+\left(\frac{2}{3}\right)\sqrt{7}\sqrt{5}m-\left(\frac{2}{3}\right)\sqrt{7}\sqrt{5}n$, $NAG_{1}=18mn+27m+9n+\left(\frac{54}{5}\right)\sqrt{5}mn+\left(\frac{9}{5}\right)\sqrt{5}m-\left(\frac{9}{5}\right)\sqrt{5}n+\left(\frac{144}{35}\right)\sqrt{7}\sqrt{5}mn+\left(\frac{24}{35}\right)\sqrt{7}\sqrt{5}m-\left(\frac{24}{35}\right)\sqrt{7}\sqrt{5}n$ and $N\chi \left(\Gamma \right)=3\sqrt{2}mn+\left(\frac{13}{2}\right)\sqrt{2}m+\left(\frac{5}{2}\right)\sqrt{2}n+8mn+\left(\frac{4}{3}\right)m-\left(\frac{4}{3}\right)n+4\sqrt{3}mn+\left(\frac{2}{3}\right)\sqrt{3}m-\left(\frac{2}{3}\right)\sqrt{3}n+\left(\frac{3}{7}\right)\sqrt{2}\sqrt{7}mn+\left(\frac{1}{14}\right)\sqrt{2}\sqrt{7}m-\left(\frac{1}{14}\right)\sqrt{2}\sqrt{7}n$.

**Figure 14:**
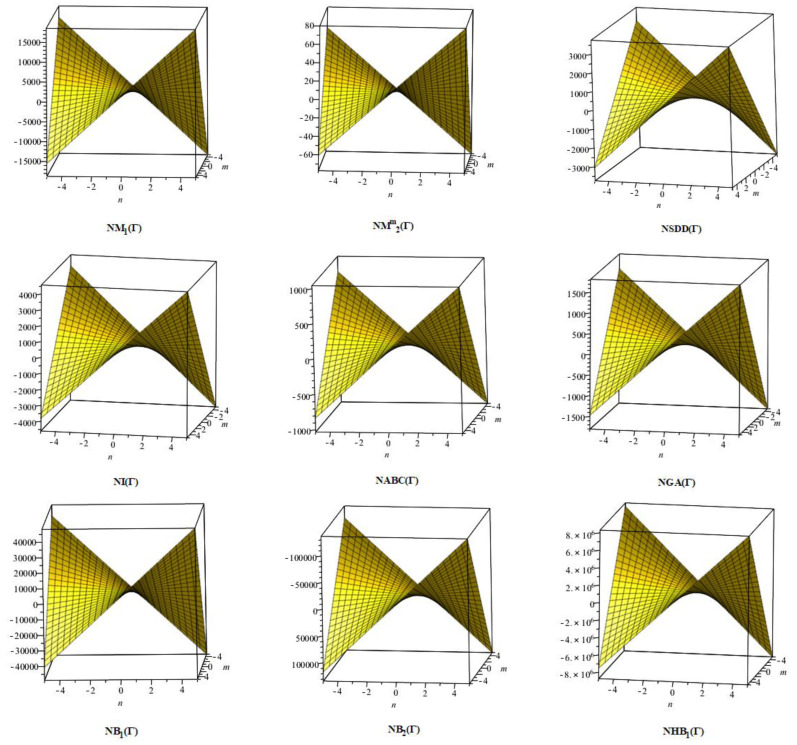
3D plots for theorem 8.

**Figure 15:**
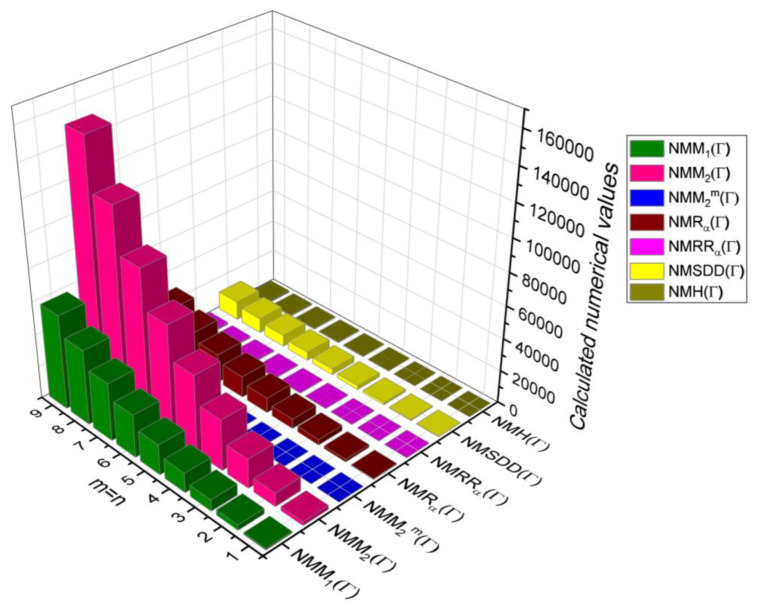
3D plots for Table 13.

**Figure 16:**
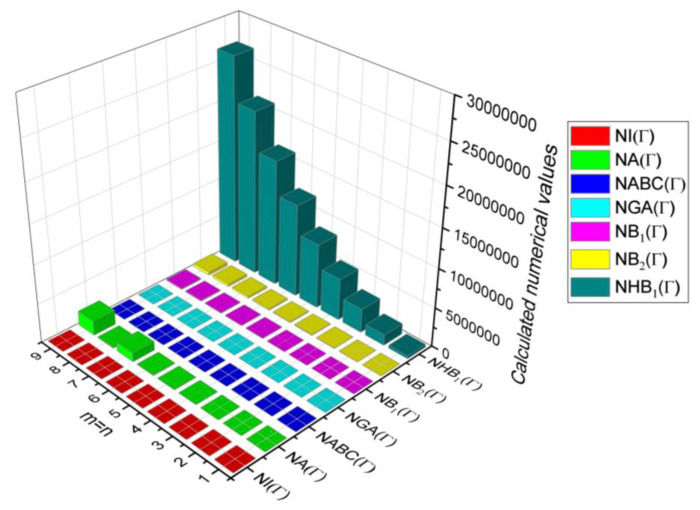
3D plots for Table 14.

**Figure 17:**
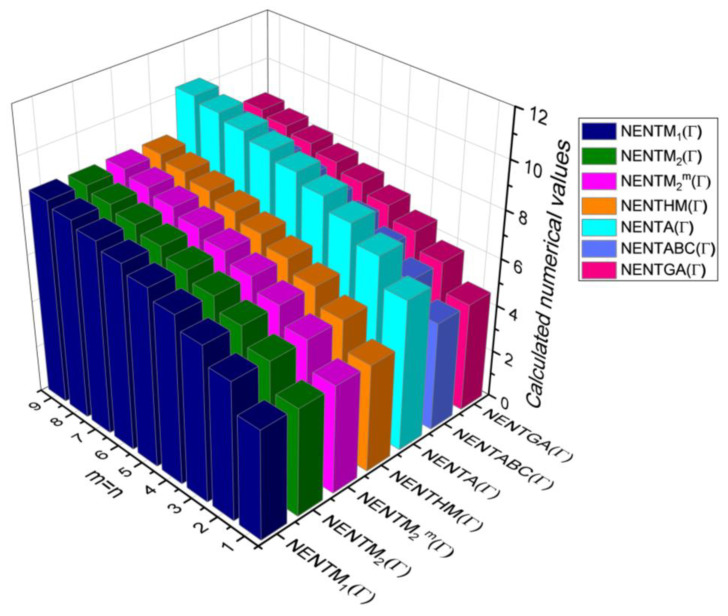
3D plots for Table 15.

**Figure 18:**
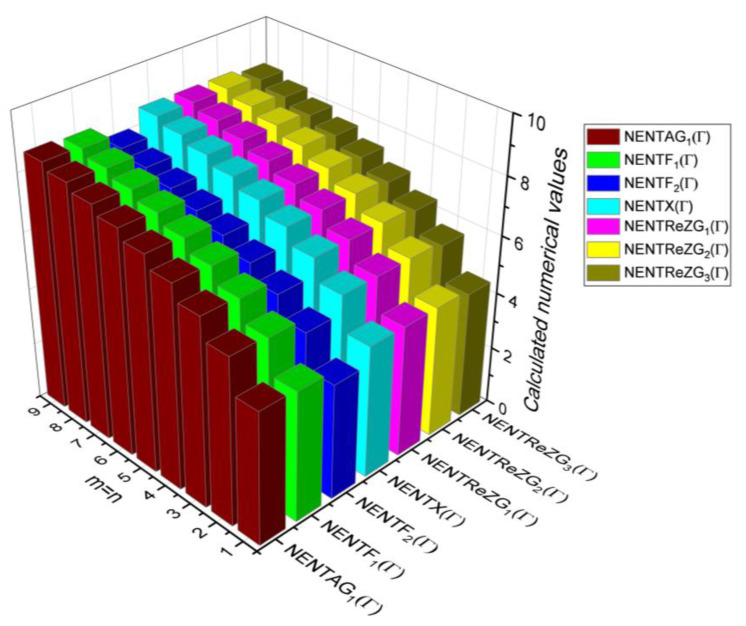
3D plots for Table 16.

In this section, the neighbourhood degree sum-based indices computed in Theorem 8 and Theorem 9 are plotted as 3D graphs to provide a visual tool for understanding the behavioural pattern of the indices for changing molecular structures. In addition, the results of Theorem 8 have also been presented as comparison plots in Figure 14 to study the behaviour of the indices with respect to each other and the molecular structure. The numerical values of the indices are computed and listed in Table 13, Table 14, Table 15 and Table 16. The numerical values are represented as 3D plots in Figure 15, Figure 16, Figure 17, and Figure 18 respectively to provide a visual understanding to the readers.

## 11. Conclusions

In this article, the closed-form analytical expressions of neighbourhood sum degree-based indices of three types of graphenes have been derived using the M-Polynomial. The computed indices are presented as individual 3D plots and comparison plots for a convenient interpretation of the mathematical expressions. The neighbourhood sum degree-based entropy measures have also been calculated for the three types of graphene structures. These indices are also visualised as 3D plots to corroborate the dependence of the indices on the underlying molecular structure. These indices have not been studied before for these structures; hence, this study is one of a kind. This study will enable future researchers to explore more topological indices for these fascinating structures.

## Figures and Tables

**Table 1:** NM-Polynomial Expressions.

S. No	Degree-Based TIs Derived from NM(Γ;𝓇,𝓽)
1.	${M_{1}\left(\Gamma \right)=D_{𝓇}+D_{𝓽}\left(NM\left(\Gamma ;𝓇,𝓽\right)\right)|}_{𝓇=𝓽=1}$
2.	${M_{2}\left(\Gamma \right)=D_{𝓇}D_{𝓽}\left(NM\left(\Gamma ;𝓇,𝓽\right)\right)|}_{𝓇=𝓽=1}$
3.	${M_{2}^{m}\left(\Gamma \right)=S_{𝓇}S_{𝓽}\left(NM\left(\Gamma ;𝓇,𝓽\right)\right)|}_{𝓇=𝓽=1}$
4.	${R_{\alpha }\left(\Gamma \right)=D_{𝓇}^{\alpha }D_{𝓽}^{\alpha }\left(NM\left(\Gamma ;𝓇,𝓽\right)\right)|}_{𝓇=𝓽=1}$
5.	${RR_{\alpha }\left(\Gamma \right)=S_{𝓇}^{\alpha }S_{𝓽}^{\alpha }\left(NM\left(\Gamma ;𝓇,𝓽\right)\right)|}_{𝓇=𝓽=1}$
6.	$SDD\left(\Gamma \right)=({D_{𝓇}S_{𝓽}+D_{𝓽}S_{𝓇})\left(NM\left(\Gamma ;𝓇,𝓽\right)\right)|}_{𝓇=𝓽=1}$
7.	$H\left(\Gamma \right)=2S_{𝓇}J{\left(NM\left(\Gamma ;𝓇,𝓽\right)\right)|}_{𝓇=1}$
8.	$I\left(\Gamma \right)=S_{𝓇}JD_{𝓇}D_{𝓽}{\left(NM\left(\Gamma ;𝓇,𝓽\right)\right)|}_{𝓇=1}$
9.	$A\left(\Gamma \right)=S_{𝓇}^{3}Q_{-2}\;JD_{𝓇}^{3}D_{𝓽}^{3}{\left(NM\left(\Gamma ;𝓇,𝓽\right)\right)|}_{𝓇=1}$
10.	$ABC\left(\Gamma \right)=D_{𝓇}^{\frac{1}{2}}\;Q_{-2}J{S_{𝓇}^{\frac{1}{2}}S_{𝓽}^{\frac{1}{2}}\left(NM\left(\Gamma ;𝓇,𝓽\right)\right)|}_{𝓇=1}$
11.	$GA\left(\Gamma \right)=2S_{𝓇}JD_{𝓇}^{\frac{1}{2}}{D_{𝓽}^{\frac{1}{2}}\left(NM\left(\Gamma ;𝓇,𝓽\right)\right)|}_{𝓇=1}$
12.	$B_{1}\left(\Gamma \right)=\left(D_{𝓇}+D_{𝓽}+2D_{𝓇}Q_{-2}J\right){\left(NM\left(\Gamma ;𝓇,𝓽\right)\right)|}_{𝓇=𝓽=1}$
13.	$B_{2}\left(\Gamma \right)=D_{𝓇}Q_{-2}J\left(D_{𝓇}+D_{𝓽}\right){\left(NM\left(\Gamma ;𝓇,𝓽\right)\right)|}_{𝓇=1}$
14.	$HB_{1}\left(\Gamma \right)=D_{𝓇}^{2}+D_{𝓽}^{2}+2D_{𝓇}^{2}Q_{-2}J+{\left.2D_{𝓇}Q_{-2}J\left(D_{𝓇}+D_{𝓽}\right)\left(NM\left(\Gamma ;𝓇,𝓽\right)\right)\right|}_{𝓇=𝓽=1}$

**Table 2:** Partition table for β-Graphene.

Edge Types	Frequency
$d_{55}$	4m+2n
$d_{57}$	4m+8
$d_{58}$	4m+4n−8
$d_{79}$	2m+4
$d_{88}$	2n−2
$d_{89}$	8m+4n−12
$d_{99}$	18mn−21m−n+10

**Table 3:** Comparison Table for Theorem 2.

m=n	$NMM_{1}\left(\Gamma \right)$	$NMM_{2}\left(\Gamma \right)$	$NMM_{2}^{m}\left(\Gamma \right)$	$NMR_{\alpha }\left(\Gamma \right)$	$NMRR_{\alpha }\left(\Gamma \right)$	NMSDD(Γ)	NMH(Γ)
1	408	1434	0.75	202.61	4.65	61.75	4.62
2	1464	5754	1.93	728.58	13.26	196.30	13.18
3	3168	12990	3.55	1578.53	25.88	402.85	25.75
4	5520	23142	5.62	2752.49	42.49	681.40	42.31
5	8520	36210	8.13	4250.45	63.10	1032.00	62.88

**Table 4:** Comparison Table for Theorem 2.

m=n	NMI(Γ)	NMA(Γ)	NMABC(Γ)	NMGA(Γ)	$NMB_{1}\left(\Gamma \right)$	$NMB_{2}\left(\Gamma \right)$	$NMHB_{1}\left(\Gamma \right)$
1	100.62	2022.83	15.30	29.79	1104	4992	509712
2	362.61	8614.42	46.61	95.48	4008	20268	2464404
3	786.59	19876.87	93.91	197.17	8712	45912	5912088
4	1372.57	35810.19	157.22	334.86	15216	81924	10852764
5	2120.55	56414.37	236.53	508.56	23520	128304	17286432

**Table 5:** Comparison Table for Theorem 3.

m=n	$NENT_{NM_{1\left(\Gamma \right)}}$	$NENT_{NM_{2\left(\Gamma \right)}}$	${NENT}_{NM_{2}^{m}\left(\Gamma \right)}$	$NENT_{{NHM}_{\left(\Gamma \right)}}$	$NENT_{{NA}_{\left(\Gamma \right)}}$	$NENT_{{NABC}_{\left(\Gamma \right)}}$	$NENT_{{NGA}_{\left(\Gamma \right)}}$
1	3.38	3.31	3.32	3.31	4.42	3.40	3.40
2	4.54	4.49	4.46	4.49	5.34	4.56	4.56
3	5.27	5.23	5.18	5.23	6.00	5.28	5.29
4	5.80	5.77	5.72	5.77	6.49	5.81	5.82
5	6.22	6.20	6.15	6.20	6.89	6.23	6.23

**Table 6:** Comparison Table for Theorem 3.

m=n	$NENT_{{NAG}_{1\left(\Gamma \right)}}$	$NENT_{{NF}_{1\left(\Gamma \right)}}$	$NENT_{{NF}_{2\left(\Gamma \right)}}$	$NENT_{{N\chi }_{\left(\Gamma \right)}}$	$NENT_{{NREZG}_{1}{}_{\left(\Gamma \right)}}$	$NENT_{{NREZG}_{2}{}_{\left(\Gamma \right)}}$	$NENT_{{NREZG}_{3}{}_{\left(\Gamma \right)}}$
1	3.40	3.31	3.08	6.80	3.69	4.39	3.21
2	4.56	4.50	4.35	8.41	4.82	5.52	4.42
3	5.29	5.24	5.13	9.35	5.53	6.22	5.18
4	5.82	5.77	5.70	10.00	6.05	6.74	5.73
5	6.23	6.20	6.13	10.51	6.47	7.16	6.17

**Table 7:** Partition Table for β- Graphyne.

Edge Types	Frequency
$d_{44}$	12m+6n
$d_{45}$	8m+4n
$d_{55}$	12mn−2m−4n
$d_{57}$	24mn+4m−4n
$d_{77}$	6mn−21m+21n

**Table 8:** Comparison Table for Theorem 5.

m=n	$NMM_{1}\left(\Gamma \right)$	$NMM_{2}\left(\Gamma \right)$	$NMM_{2}^{m}\left(\Gamma \right)$	${NMR}_{\alpha }\left(\Gamma \right)$	${NMRR}_{\alpha }\left(\Gamma \right)$	NMSDD(Γ)	NMH(Γ)
1	684	1812	2.77	339.65	13.30	135.34	13.22
2	2352	6492	8.12	1167.27	41.22	444.17	40.96
3	5004	14040	16.05	2482.87	83.78	926.49	83.21
4	8640	24456	26.55	4286.44	140.95	1582.29	139.98
5	13260	37740	39.63	6577.98	212.77	2411.57	211.26

**Table 9:** Comparison Table for Theorem 5.

m=n	NMI(Γ)	NMA(Γ)	NMABC(Γ)	NMGA(Γ)	$NMB_{1}\left(\Gamma \right)$	$NMB_{2}\left(\Gamma \right)$	${NMHB}_{1}\left(\Gamma \right)$
1	168.67	2241.83	37.32	65.59	1788	5988	326316
2	579.33	8091.08	119.80	214.51	6192	21672	1253976
3	1232.00	17547.76	247.46	446.77	13212	47052	2782980
4	2126.67	30611.88	420.29	762.34	22848	82128	4913328
5	3263.33	47283.42	638.29	1161.24	35100	126900	7645020

**Table 10:** Comparison Table for Theorem 6.

m=n	$NENT_{M_{1\left(\Gamma \right)}}$	$NENT_{M_{2\left(\Gamma \right)}}$	$NENT_{M_{2}{{}^{m}}_{\left(\Gamma \right)}}$	$NENT_{HM_{\left(\Gamma \right)}}\;$	$NENT_{A_{\left(\Gamma \right)}}$	$NENT_{ABC_{\left(\Gamma \right)}}$	$NENT_{GA_{\left(\Gamma \right)}}$
1	4.17	4.12	4.12	2.61	5.90	4.19	4.19
2	5.36	5.32	5.31	4.52	7.08	5.37	5.38
3	6.10	6.06	6.05	5.52	7.81	6.11	6.11
4	6.63	6.60	6.59	6.19	8.34	6.64	6.64
5	7.05	7.03	7.02	6.70	8.77	7.06	7.06

**Table 11:** Comparison Table for Theorem 6.

m=n	$NENT_{AG_{1\left(\Gamma \right)}}$	$NENT_{F_{1\left(\Gamma \right)}}$	$NENT_{F_{2\left(\Gamma \right)}}$	$NENT_{{N\chi }_{\left(\Gamma \right)}}$	$NENT_{REZG_{1}{}_{\left(\Gamma \right)}}$	$NENT_{REZG_{2}{}_{\left(\Gamma \right)}}$	$NENT_{REZG_{3}{}_{\left(\Gamma \right)}}$
1	4.19	4.33	3.62	4.19	4.17	4.17	4.04
2	5.38	5.55	4.85	5.37	5.36	5.36	5.26
3	6.11	6.30	5.60	6.11	6.10	6.10	6.01
4	6.64	6.85	6.15	6.64	6.63	6.63	6.55
5	7.06	7.28	6.58	7.06	7.05	7.05	6.98

**Table 12:** Partition Table for β-Graphdiyne.

Edge Types	Frequency
$d_{44}$	12mn+26m+10m
$d_{45}$	24mn+4m−4n
$d_{57}$	24mn+4m−4n
$d_{77}$	6mn+m−n

**Table 13:** Comparison Table for Theorem 8.

m=n	$NMM_{1}\left(\Gamma \right)$	$NMM_{2}\left(\Gamma \right)$	$NMM_{2}^{m}\left(\Gamma \right)$	$NMR_{\alpha }\left(\Gamma \right)$	$NMRR_{\alpha }\left(\Gamma \right)$	NMSDD(Γ)	NMH(Γ)
1	972	2382	5.01	483.32	22.28	207.94	22.19
2	3312	8376	15.53	1645.27	71.12	687.77	70.76
3	7020	17982	31.57	3485.85	146.52	1439.49	145.71
4	12096	31200	53.13	6005.07	248.48	2463.09	247.05
5	18540	48030	80.20	9202.93	377.01	3758.57	374.76

**Table 14:** Comparison Table for Theorem 8.

m=n	NMI(Γ)	NMA(Γ)	NMABC(Γ)	NMGA(Γ)	$NMB_{1}\left(\Gamma \right)$	$NMB_{2}\left(\Gamma \right)$	$NMHB_{1}\left(\Gamma \right)$
1	240.33	2907.49	59.39	101.52	2508	6744	365784
2	817.33	10264.64	193.47	334.07	8592	23520	1380192
3	1731.00	22071.43	402.24	697.66	18252	50328	3043224
4	2981.33	38327.88	685.72	1192.25	31488	87168	5354880
5	4568.33	59033.98	1043.85	1817.89	48300	134040	8315160

**Table 15:** Comparison Table for Theorem 9.

m=n	$NENT_{M_{1\left(\Gamma \right)}}$	$NENT_{M_{2\left(\Gamma \right)}}$	$NENT_{M_{2}{{}^{m}}_{\left(\Gamma \right)}}$	$NENT_{HM_{\left(\Gamma \right)}}\;$	$NENT_{A_{\left(\Gamma \right)}}$	$NENT_{ABC_{\left(\Gamma \right)}}$	$NENT_{GA_{\left(\Gamma \right)}}$
1	4.61	4.54	4.57	4.54	6.37	4.62	4.63
2	5.80	5.74	5.76	5.74	7.54	5.81	5.82
3	6.53	6.48	6.49	6.47	8.27	6.55	6.55
4	7.07	7.01	7.03	7.01	8.81	7.09	7.09
5	7.49	7.44	7.45	7.44	9.23	7.51	7.51

**Table 16:** Comparison Table for Theorem 9.

m=n	$NENT_{AG_{1\left(\Gamma \right)}}$	$NENT_{F_{1\left(\Gamma \right)}}$	$NENT_{F_{2\left(\Gamma \right)}}$	$NENT_{\chi_{\left(\Gamma \right)}}$	$NENT_{REZG_{1}{}_{\left(\Gamma \right)}}$	$NENT_{REZG_{2}{}_{\left(\Gamma \right)}}$	$NENT_{REZG_{3}{}_{\left(\Gamma \right)}}$
1	4.63	4.54	4.03	4.62	4.61	4.61	4.44
2	5.82	5.73	5.22	5.81	5.80	5.80	5.64
3	6.55	6.47	5.96	6.55	6.54	6.54	6.38
4	7.09	7.01	6.50	7.09	7.07	7.07	6.92
5	7.51	7.43	6.93	7.51	7.50	7.49	7.35

## Data Availability

No data associated in the manuscript.
